# Genome-Wide Analysis of the AP2/ERF Family in *Eucalyptus grandis*: An Intriguing Over-Representation of Stress-Responsive *DREB1/CBF* Genes

**DOI:** 10.1371/journal.pone.0121041

**Published:** 2015-04-07

**Authors:** P. B. Cao, S. Azar, H. SanClemente, F. Mounet, C. Dunand, G. Marque, C. Marque, C. Teulières

**Affiliations:** 1 Université de Toulouse, UPS, UMR 5546, LRSV, 24 Chemin de Borde Rouge, Auzeville, BP 42617 31326, Castanet-Tolosan, France; 2 CNRS, UMR 5546, LRSV, 24 Chemin de Borde Rouge, Auzeville, BP 42617 31326, Castanet-Tolosan, France; Chinese Academy of Sciences, CHINA

## Abstract

**Background:**

The AP2/ERF family includes a large number of developmentally and physiologically important transcription factors sharing an AP2 DNA-binding domain. Among them *DREB1/CBF* and *DREB2* factors are known as master regulators respectively of cold and heat/osmotic stress responses.

**Experimental Approaches:**

The manual annotation of AP2/ERF family from *Eucalyptus grandis*, *Malus*, *Populus* and *Vitis* genomes allowed a complete phylogenetic study for comparing the structure of this family in woody species and the model *Arabidopsis thaliana*. Expression profiles of the whole groups of *EgrDREB1* and *EgrDREB2* were investigated through RNAseq database survey and RT-qPCR analyses.

**Results:**

The structure and the size of the AP2/ERF family show a global conservation for the plant species under comparison. In addition to an expansion of the ERF subfamily, the tree genomes mainly differ with respect to the group representation within the subfamilies. With regard to the *E*. *grandis DREB* subfamily, an obvious feature is the presence of 17 *DREB1/CBF* genes, the maximum reported to date for dicotyledons. In contrast, only six *DREB2* have been identified, which is similar to the other plants species under study, except for *Malus*. All the *DREB1/CBF* and *DREB2* genes from *E*. *grandis* are expressed in at least one condition and all are heat-responsive. Regulation by cold and drought depends on the genes but is not specific of one group; DREB1/CBF group is more cold-inducible than DREB2 which is mainly drought responsive.

**Conclusion:**

These features suggest that the dramatic expansion of the *DREB1/CBF* group might be related to the adaptation of this evergreen tree to climate changes when it expanded in Australia.

## Introduction

Native to Australia, *Eucalyptus* tree species are among the fastest growing woody plants in the world and represent about 8% of all planted forest with over 18 million hectares grown in 90 countries [1]. Over the last few decades, *Eucalyptus* trees became one of the main wood fiber crops and a cost-effective source of lignocellulosic biomass for energy production. Market favorites for pulpwood are *E*. *grandis*, *E*. *urophylla* and their hybrids, grown in tropical and subtropical regions, and *E*. *globulus*, planted in temperate regions. Frost tolerance is limiting their expansion in temperate climates, such as in Southern Europe or Southeastern USA [2]. Being evergreen and evergrowing, the *Eucalyptus* trees are exposed to cold while growing and all the organs may have to cope with frost.

The 605 Mb *E*. *grandis* genome sequence released in January 2011 including about 96% of the expressed gene loci [3] now allows genome-wide investigations. In the present study, this resource was surveyed to manually annotate the AP2/ERF (APETALA2/Ethylene-Responsive element binding Factor) family which is characterized by a highly conserved AP2-DNA binding domain [4, 5]. This transcription factor family is known to play a key role in various developmental and adaptive processes in plants, in particular through biotic and abiotic stress response [6, 7]. Ranging between 132 and 292 genes, this family is one of the largest groups of transcription factors [8, 9] and has been classified into subfamilies [10]. The AP2 subfamily contains proteins exhibiting two AP2/ERF domains and known to be involved in the regulation of developmental processes [11–13]. The RAV (**R**elated to **A**BI3/**V**P1) proteins which contain two different DNA-binding domains (AP2/ERF and B3) are regulated by ethylene [14] or brassinosteroids [15] and also contribute to biotic and abiotic stress responses [16]. Exhibiting only one AP2/ERF domain, the ERF and DREB (**D**ehydration **R**esponsive **E**lement **B**inding) proteins are key regulators of plant responses to biotic and abiotic stresses [17, 18]. The ERF subfamily includes a large number of proteins binding to the GCC box [19], an ethylene responsive element located in the promoter of pathogenesis-related genes [20]. The DREB factors which recognize the cis-acting sequences CRT (**C**-**R**epea**t**) or DRE (**D**ehydration **R**esponsive **E**lement) located in the promoters of abiotic stress responsive genes [10]. The DREB subfamily has been divided into six groups (A1-A6) which includes the A1 and A2 groups, commonly known as *DREB1/CBF* and *DREB2* [10].The *DREB1/CBF* genes are mainly known as cold-responsive whereas most of the *DREB2* genes have generally been reported as responsive to water stress or heat-shock. However, there is a growing evidence that the stress regulation of *DREB1/CBF* and *DREB2* genes differs according to the plant species [6, 7],[21], [22, 23]. The demonstration of a functional role of *Eucalyptus CBF* genes in abiotic stress tolerance was achieved in our hands through overexpression in the hybrid *E*. *urophylla x E*. *grandis* [24]

For the first time, the paper presents a comparison of phylogenetic structure of *AP2/ERF* families based on manually annotated sequences from four woody plants (*Eucalyptus*, *Populus*, *Malus* and *Vitis*) and *Arabidopsis*. Since the number of *CBF* gene copies was found to be genetically related to stress tolerance [25], the present study may provide new information on the distinct evolution of these species to cope with different environments. The second part of this paper focuses on the *DREB*1/*CBF* and *DREB*2 which are the most described groups with regard to stress response. Interestingly, the comparison of *DREB* groups from woody species reveals an over-representation of *DREB1/CBF* members in *E*. *grandis* genome, together with a moderate number of *DREB2* genes. In addition, gene expression of all the members of *DREB*1/*CBF* and *DREB*2 groups in response to various stresses or in different tissues is presented. The data, which raise the question about specificity of these groups in stress response (cold/heat/drought), are discussed in the context of the adaptive evolution of *Eucalyptus*.

## Materials and Methods

### Source of genome databases

The annotated *E*. *grandis* genome sequence (V1.1) is available at Phytozome website (http://www.phytozome.net/eucalyptus.php). The genomic sequences of *A*. *thaliana* AP2/ERF family were down-loaded from the DATF (Database of *Arabidopsis* Transcription Factors) database website (http://datf.cbi.pku.edu.cn) [26]. The genomic sequences of the *AP2/ERF* family genes from *Vitis*, *Populus* and *Malus* were obtained from the Phytozome website and from published data [27], [28], [29], [23]. For each of these woody species, the manual annotation of DREB1/CBF and DREB2 groups was based both on the search of conserved binding domains and the analysis of the complete protein sequence.

Finally, the RNAseq database (http://anjie.bi.up.ac.za/eucGenIE/EucGenIE.html) was used for investigating *DREB1* and *DREB2* gene expression in different tissues. Raw expression data were obtained from the *Eucalyptus* genome integrative explorer (http://eucgenie.bi.up.ac.za) [30]. Heat map and hierarchical clustering of genes expression profiles were performed by using expander software (ACGT, http://www.cs.tau.ac.il).

### Identification and annotation of *E*. *grandis* AP2/ERF proteins

Different approaches were used for the identification and annotation of all *E*. *grandis* AP2/ERF family members. First, the PS51032 domain of Prosite and alternatively the *Arabidopsis* AP2/ERF domains were used as query sequences against the whole translated genome of *E*. *grandis*. Because some predicted *E*. *grandis* AP2/ERF proteins were not detected in the proteome of *Arabidopsis*, *Vitis*, *Malus* or *Populus*, a manual annotation of the *E*. *grandis* genomic sequences was performed by combining a search of homology (http://blast.ncbi.nlm.nih.gov/), and gene prediction (http://augustus.gobics.de/). In addition, a search for new putative paralogs was performed using SCIPIO, an alignment-based tool [31]. Previously predicted and newly discovered sequences were scanned for the presence of characteristic domains (AP2 and B3) using PROSITE patterns and profiles (http://www.expasy.org/prosite/) or Pfam (http://pfam.sanger.ac.uk/). If a domain (or part of) was missing, a manual annotation was performed to complete or correct the predicted sequence. Finally, the structure and genome localization of each gene was determined by using the SCIPIO program on the resulting exhaustive set of EgrAP2/ERF family members.

### Manual annotation and mapping of *EgrDREB1/CBF* genes

In addition to the automatically predicted sequences, the presence of the CBF signatures (PKK/RPAGRxKFxETRHP and DSAWR) [32] was checked using DiALign on the complete translated *E*. *grandis* genome. Following the EUCAGEN consortium recommendation, the *EgrCBF* sequences were named according to their relative position on the classified scaffolds (1 to 11).Then, the cDNA sequences of four previously described *E*. *gunnii CBFs* [33] were compared to the *E*. *grandis* genome by using the SPLIGN program proposed on the NCBI website for annotating the genomes of higher eukaryotes [34]. At this stage, the available *E*. *grandis* genome may still contain a number of sequencing errors leading to falsely assigned codons [35]. Indeed, some of the predicted sequences appeared to be incorrect since they exhibit wrong positions for the start and/or stop codons or unlikely intronic sequences, compared to typical CBF sequences [36], [37]. To validate the hypothesis of erroneous predictions, new sequencing experiments were performed on DNA and cDNA sequences and analyzed to propose curate sequences. Leaving out the synonym “DREB1” for simplification, the validated EgrCBF proteins were classified according to the *E*. *gunnii* orthologs by using the EMBOSS Needle program.

### Phylogenetic tree construction

Multiple alignments were prepared using ClustalW [38] using default parameters (gap opening penalty = 10, gap extension penalty = 0.1) and further inspected and visually adjusted with BioEdit [39]. The resulting alignments of complete protein sequences were used in MEGA (version 5) [40] for the construction of phylogenetic trees based on Maximum Likelihood according to Jones-Taylor-Thornton model with uniform rates among sites and complete deletion of gaps/missing data. Bootstrap values were calculated from 100 replicates [41].

### Plant material and stress treatments for gene expression analysis

*E*. *grandis* plants grown from commercial seeds were obtained from Australia (www.b-and-t-world-seeds.com) and *E*. *gunnii x E*. *dalrympleana* (clone 208) hybrid plants issued from cuttings were provided by FCBA (France). One year-old plants from the two species were cultivated under standard conditions of 25°C day/22°C night, 16 h day-length and 115 μmoles.m2.s-1 light intensity (Lumilux Daylight 58W, Osram, München, Germany). The relative humidity was kept at approximately 80% and plants were watered as needed.

To quantify *CBF* and *DREB2* transcription in response to stressful conditions, the first experiment was conducted on *E*. *gunnii x E*. *dalrympleana* plants directly transferred from 22°C to 4°C for a 2h-light period followed by a 6h-dark phase, according to previous experiments in our hands [42]. The control plants were kept at 25°C/light for 2h and then at 25°C/dark for 6h. Each sample was composed of all the leaves harvested from two individual plants and pooled for RNA extraction. Transcript quantification was performed on three independent samples (biological replicates).

According to our previous data on *Eucalyptus* simplified material [43], the second experiment was performed on detached leaves which were submitted to different stress conditions. Thirty leaves (from 10 individual *E*. *grandis* plants) were harvested to form three independent samples of ten leaves (biological replicates). The samples were kept for 2h under light followed by a 6h-dark phase, before RNA extraction (on the pool of 10 leaves). For control, heat and cold stress, the petioles of the detached leaves were kept immersed in a liquid medium (¼ MS) in a culture chamber. The stressed samples were submitted either to a cold-shock at 4°C or to a heat-shock at 45°C, while the control samples were kept at 25°C. For drought stress, the detached leaves were left on Whatman paper inside an incubator at 25°C using the same enlightening protocol.

### Quantification of gene expression through Real-Time PCR (RT-qPCR)

RNA extraction was performed using a modified protocol of the SV Total RNA Isolation System (Promega, France). The modification consisted in complementing the NTES buffer (0.1 M NaCl; 0.01 M Tris/HCl; pH 7.5; 1 mM EDTA) with 2% polyvinylpyrrolidone (PVP) and 2% 2-mercaptoethanol [44]. Also an additional washing step was included to improve the RNA purity. Using SuperScript II and random primers (Invitrogen, France), cDNA was produced from 3 μg of extracted RNA according to the manufacturer’s instructions. Based on the corrected *EgrCBF* annotation, oligonucleotide primers were designed to specifically amplify the different members of the family (S1 Table).

Due to the very high similarity between the Egr*CBF* genes, the design of specific primers for an efficient RT-qPCR amplification of each paralog was challenging, and additional control steps were needed to confirm that the PCR amplification is specific. To check sequence homology, each primer sequence was blasted against both *E*. *grandis* and *E*. *gunnii* genomes. The use of DNA calculator (http://www.sigma-genosys.com/calc/DNACalc.asp) provided a technical validation for each primer. The final step for controlling the specificity of amplification was the analysis of the PCR products by gel-electrophoresis and dissociation curves.

The RT-qPCR reactions were performed in 10 μL of SYBR Green Master mix (Applied Biosystems), with 100 ng of cDNA and 300 nM of each primer. Three replicates of each cDNA were run in an ABI PRISM 7900HT 295 Sequence Detection System (Applied Biosciences, France) using a program including a first step (50°C for 2 min and 95°C for 10 min) followed by 40 cycles (95°C/15 s and 61°C/1 min). Serine/threonine-protein phosphatase 2A PP2A1 (Eucgr.B03386) and PP2A3 (Eucgr.B03031), IDH (Eucgr.F02901), SAND (Eucgr.B02502) and EF1A (Eucgr.B02473) were used together as reference genes for the normalization of RNA steady-state level, and the relative changes in gene expression were quantified using the 2-ΔΔ Ct method [45].

The relative transcript abundances are presented as the mean expression Log_10_ values for the experiments based on whole plants. For the experiments on detached leaves, the relative expression values are directly presented as Log_10_ of induction rate for each stress condition in comparison to the control. In both cases, the experiments were performed on three independent biological replicates (each sample composed of material from individual plants). The mean values were analyzed for significance using the statistical T-test (p value = 0.05).

## Results

### Annotation and structure of the AP2/ERF transcription factor family from *E*. *grandis* in comparison with other plant species

From the 36376 loci encoding putative proteins in the *E*. *grandis* genome [3, 46], 209 were identified as AP2/ERF-like proteins based on the presence of an AP2 DNA-binding domain. When several sequences were predicted at the same genomic locus, the selection of a unique sequence was based on the highest similarity with previously described orthologs. Some predicted genes were discarded because they lack a complete AP2 domain or have a very different ORF compared to the AP2/ERF sequences from other plant species. The phylogenetic trees corresponding to each subfamily in *A*. *thaliana* and *E*. *grandis* are shown in S1, S2, S3 and S4 Figs. The analysis of key residues together with the phylogenetic trees allowed classifying the AP2/ERF members from *E*. *grandis* into a soloist and the four typical subfamilies, showing a conserved family structure for the plant species under study.

After the release of the *Malus* genome sequence, it was reported [3, 23] that the *AP2/ERF* family contains 259 members grouped according to the Nakano classification [47]. However, based on our manual annotation, four additional *CBF* sequences were identified and 21 sequences were discarded, (mainly within AP2 subfamily) because they were found as doublets (12 sequences overlapping with other *AP2/ERF* at the same locus) or exhibit an incomplete AP2 domain (9 sequences). The *Malus* AP2 subfamily size (51 members) predicted by Girardi and co-workers [23] is significantly smaller following our annotation, since 11 sequences have been discarded and 12 other sequences are proposed as *AP2-like* genes (because they exhibit high homology with *AP2* genes but contain only one AP2 domain). Finally, the 242 *AP2/ERF* members from *Malus* were grouped in our hands according to Sakuma classification [10], which allow the structure of the AP2/ERF family to be compared to the four other species under study (Table 1).

**Table 1:** Comparison of AP2/ERF family composition (number of genes in subfamilies and groups) between *Arabidopsis thaliana Vitis vinifera*, *Populus trichocarpa*, *Eucalyptus grandis* and *Malus domestica*.

AP2/ERF family	Groups	*A*. *thaliana*	*V*.*vinifera*	*P*. *trichocarpa*	*E*. *grandis*	*M*. *domestica*
DREB subfamily	A1	6	7	6	17	5 + 1p
	A2	8	5	8	6	14
	A3	1	0	2	1	4
	A4	16	15	26	16	18
	A5	16	8	14	17	13
	A6	10	5	11	7	9
	**Subtotal**	**57**	**40**	**67**	**61 + 3p**	**63 +1p**
ERF subfamily	B1	15	11	19	12	19
	B2	5	3	6	3	7
	B3	18	41	35	65	45
	B4	7	3	7	10	13
	B5	8	4	8	5	12
	B6	12	21	26	13	30
	**Subtotal**	**65**	**83**	**101**	**103 + 5p**	**126**
AP2 subfamily		**18**	**20**	**26**	**21**	**29 + 11 like**
RAV subfamily		**6**	**6**	**5**	**6 +2p**	**6**
Soloist		**1**	**1**	**1**	**1**	**6**
**Total**		**147**	**150**	**200**	**202**	**242**

Pseudogenes are indicated as “p”.

**Fig 1:**
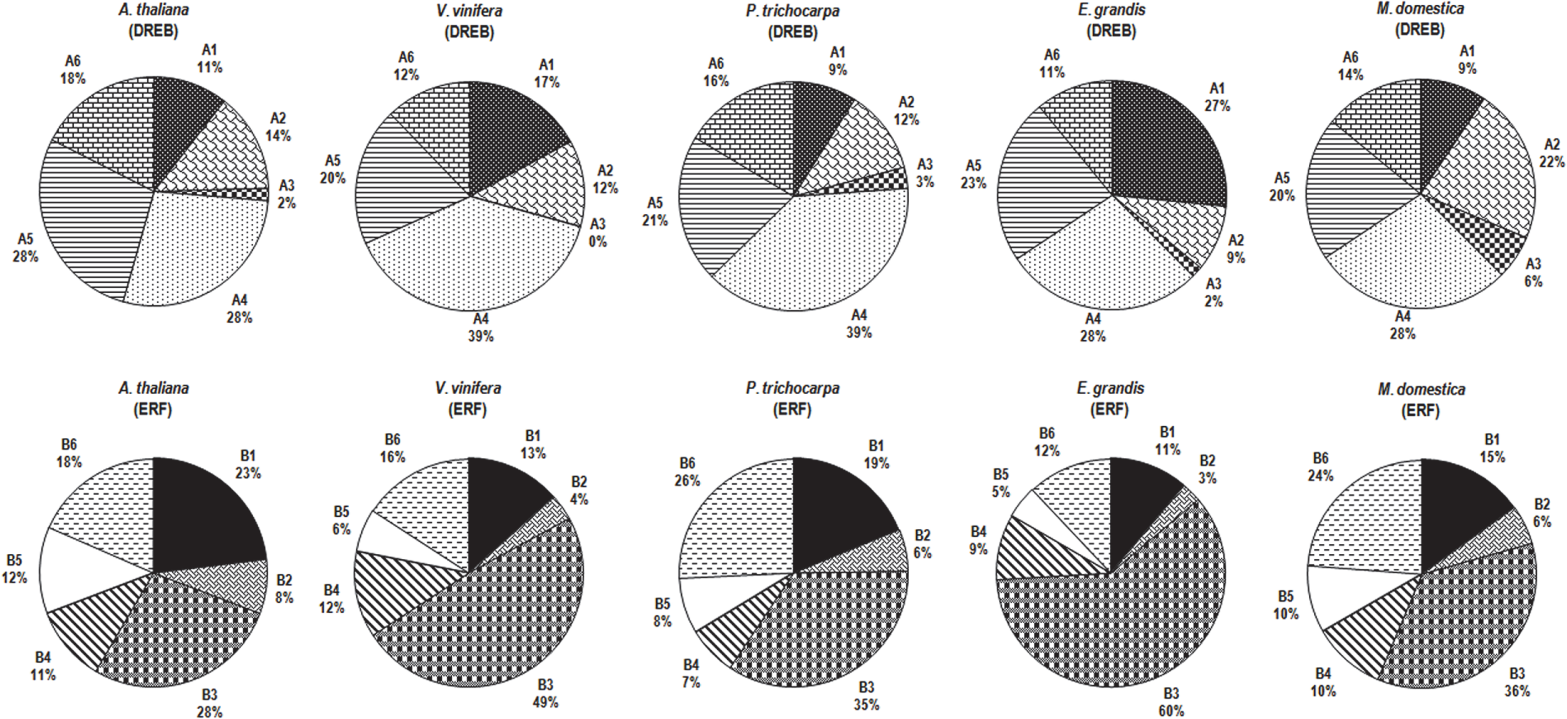
Distribution of genes within DREB and ERF subfamilies from *Arabidopsis thaliana*, *Vitis vinifera*, *Populus trichocarpa Eucalyptus grandis* and *Malus domestica*. Pie charts depicting as sectors the relative size of each group A1-A6 and B1-B6 for respectively the DREB and ERF subfamilies. Each group is presented as a percentage of the number of corresponding DREB or ERF members, as indicated in bold in Table 1.

The AP2/ERF family of *E*. *grandis* is medium-sized like in *Populus*, smaller than in *Malus* but higher than in *Vitis* and *Arabidopsis*. These plant species exhibit a similar size for the AP2 and RAV subfamilies and share a highly conserved soloist sequence, which is strongly divergent from the other AP2/ERF proteins and was only duplicated in *Malus* [23]. ERF, the most represented subfamily in the five species, exhibits an expansion in the tree genomes. With 108 genes, the *E*. *grandis* ERF subfamily represents 53.5% of the whole AP2/ERF family, which is in the same range that the other woody species (50.5% for *Populus*, 52% for *Malus* and 55.7% for *Vitis*). Within the ERF subfamily, the distribution of proteins among the B1-B6 groups is similar in *Eucalyptus* and *Vitis* but differs from the distribution in the three other species (Fig 1). Of particular note is the B3 group which with 65 members (60% of ERF subfamily) is strongly over-represented in *Eucalyptus* (Table 1 and Fig 1). In contrast to the ERF expansion, tree DREB subfamilies exhibit a similar size compared to *Arabidopsis* genome.

According to the literature, the *Malus DREB* subfamily contains various numbers of *DREB1*/*CBF* genes (2–3) and *DREB2* genes (13–16) [29], [23]. In our hands, the analysis of conserved domains and complete protein sequences led to identify six CBF and 14 DREB2 factors. For *Populus*, 17 or 18 *DREB2* (*A2*) genes have been reported [48, 49]. However, the alignment of the DREB2 protein sequences from the species under study showed that 10 of these 18 *Populus* DREB2 sequences cluster together and are very different from the other A2 factors (shorter sequence and different key residues), especially they do not exhibit the CMIV domain (motif 7) characteristic of the A2 group [50]. Moreover, when we blasted these 10 sequences against the NCBI databases, they were found to be closely related to B6 genes from *Arabidopsis* and *Vitis*. Therefore, we considered for further analyses only the eight remaining Pt*DREB2* (A2) genes for *Populus*. For *Vitis*, one Vv*DREB2* gene was identified in addition to the four members previously reported in the literature [27].

The resulting annotation data allow comparing the *DREB* subfamily (size and representation in %) for the five species under study (Table 1 and Fig 1). The subfamily size in *Eucalyptus* (64 genes, 31.7%) is similar to *Populus* (33.5%), but larger than in *Malus* (26%) and *Vitis* (26.6%). Within the subfamily, A4 is always the largest group (15 to 26 genes) but the distribution of the groups depends on the plant species (Fig 1). Interestingly, the A1 group (*DREB1/CBF*) is much more prominent in *Eucalyptus* (17 genes corresponding to 27% of the *DREB* subfamily) compared to the other species for which the six or seven genes correspond to 9% (*Populus* and *Malus*), 11% (*Arabidopsis*) and 18% (*Vitis*). In contrast, the *Eucalyptus* A2 group (six genes), similar to *Vitis* group (five genes, 12.5%), is smaller than in *Populus* and *Arabidopsis* (eight genes, respectively 12 and 14%) and above all in *Malus* (14 genes, 22% of *DREB* subfamily). Taken together this means that the tree species are similar with respect to the total number of *DREB* genes, but differ in their distribution within the groups especially in the number of genes in the A1 group, which is three times higher in *Eucalyptus* than in *Populus* and *Malus*. These data strongly suggest that the high number of *DREB1/CBF*s identified in the *E*. *grandis* genome is not a common feature in the tree species under study.

### Annotation, classification and mapping of Egr*CBF* genes

**Fig 2:**
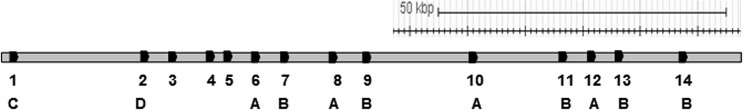
Schematic representation of the Egr*CBF* gene cluster on scaffold 1. The numbers correspond to *EgrCBF*1-14. The letters refer to *EguCBF1*A-, B-, C- or D-like, based on the percentage of identity between CBFs from *E*. *grandis* and *E*. *gunnii* (except for Egr*CBF*3, 4 and 5 unlabelled genes) (Table 3).

The 16 *CBF* sequences predicted by the Phytozome automatic annotation for *E*. *grandis* were then further investigated on the basis of their specific features, such as the presence of “signature sequences” flanking the AP2 domain and the absence of intronic sequence. An additional *EgrCBF* sequence was identified, when the four *EguCBF* sequences, previously isolated from *E*. *gunnii*, were mapped against the *E*. *grandis* genome. According to their relative position on the classified scaffolds (1 to 11), the 17 orthologs of the *E*. *grandis* genome were annotated as *EgrCBF1* to *EgrCBF*17. Very interestingly, 14 *EgrCBF* genes (1–14) are located on the scaffold 1, forming a cluster in a region of about 117 Kb (from 38661043 to 38777958) which does not contain any other gene (Fig 2). Only *EgrCBF*15, 16 and 17 are located on different loci, *EgrCBF15* on scaffold 4 and both *EgrCBF*16 and 17 on scaffold 5.

For the present work, a manual curation was required in particular for three genes (*EgrCBF3*-4-5) that were incorrectly predicted by the Phytozome automatic annotation. For both *EgrCBF4-5* genes, predicted to have an intronic sequence, the translated sequence looked like a typical *CBF* gene. The confirmation of the absence of intron for the two genes was provided by comparing the sequences amplified from cDNA and genomic DNA. In addition, the proposed *EgrCBF4* sequence includes a start-ATG codon at the 768 position (based on position 1 of the currently annotated sequence). The originally predicted *EgrCBF*3 also lacked a start-ATG codon at the normal position for a typical *CBF* sequence. Then the comparison of newly sequenced *EgrCBF3* gene and *EguCBF* orthologs confirmed the presence of the four nucleotides at the positions 7–10 which are missing in the Phytozome prediction. The *EgrCBF3* sequence could also be isolated from cDNA, showing that *EgrCBF3* can be transcribed but, because lacking start-ATG codon, it should not be translated.

The EgrCBF group can be divided into subgroups according to identity (Table 2). The most similar sequences, which share at least 90% of identical residues, correspond to three different subgroups: EgrCBF6-8-10-12, EgrCBF7-9-11-13-14, and EgrCBF16-17. Considering this very high identity level, the corresponding sequences are likely the result from duplication events which occurred more recently than those producing the other paralogs (72 to 83%). When the EgrCBF sequences were compared to the EguCBF1A-B-C-D previously isolated from *E*. *gunnii*, 11 sequences were found to exhibit a spectacularly high identity (from 91 to 99%, Table 3). While EgrCBF1 and EgrCBF2 are the closest orthologs of respectively EguCBF1C and D, the two main subgroups (EgrCBF6-8-10-12 and EgrCBF7-9-11-13-14) correspond respectively to EguCBF1A and EguCBF1B, and therefore named CBFA- and B-like.

**Table 2:** Pairwise sequence comparison (% of identity) between EgrCBF proteins from *E*. *grandis*.

	**CBF1**																
**CBF 1**	100	**CBF2**													** **		
**CBF 2**	**76**	100	**CBF3**	** **													
**CBF3**	56	69	100	**CBF4**													
**CBF 4**	67	**83**	**74**	100	**CBF5**												
**CBF 5**	56	68	60	**72**	100	**CBF6**											
**CBF 6**	**77**	**77**	60	**76**	61	100	**CBF7**	** **		** **							
**CBF 7**	**74**	**73**	56	**72**	59	80	100	**CBF8**	** **					** **			
**CBF 8**	**78**	**78**	61	**76**	63	**97**	80	100	**CBF9**			** **					
**CBF 9**	**76**	**75**	57	**74**	59	80	**93**	81	100	**CBF10**			** **				
**CBF 10**	**74**	**74**	59	**74**	60	**90**	78	**91**	79	100	**CBF11**						
**CBF 11**	**74**	**74**	56	**71**	57	78	**92**	79	**95**	76	100	**CBF12**					
**CBF 12**	**76**	**76**	62	**75**	60	**91**	79	**93**	80	**92**	79	100	**CBF 13**				
**CBF 13**	**75**	**74**	58	**74**	59	81	**96**	82	**97**	79	**95**	80	100	**CBF 14**			
**CBF 14**	**76**	**75**	58	**74**	59	81	**95**	82	**97**	79	**96**	80	**98**	100	**CBF15**		
**CBF 15**	49	46	41	44	35	48	44	46	46	45	45	47	43	46	100	**CBF16**	
**CBF 16**	42	38	31	39	30	38	38	40	39	37	40	38	40	39	47	100	**CBF17**
**CBF 17**	42	39	32	39	29	40	38	41	40	38	41	40	40	39	47	**92**	100

Bolded numbers correspond to the most identical sequences (>70% identity).

**Table 3:** Pairwise sequence comparison (% of identity) between EgrCBF and EguCBF proteins respectively from *E*. *grandis* and *E*. *gunnii*.

	EgrCBF1	EgrCBF2	EgrCBF3	EgrCBF4	EgrCBF5	EgrCBF6	EgrCBF7	EgrCBF8	EgrCBF9	EgrCBF10	EgrCBF11	EgrCBF12	EgrCBF 13	EgrCBF 14	EgrCBF15	EgrCBF16	EgrCBF17
EguCBF A	75	**75**	59	75	60	95	79	95	80	91	78	93	80	81	46	39	41
EguCBF B	76	**75**	57	73	58	81	95	82	98	80	96	81	98	99	46	40	40
EguCBF C	95	**73**	55	66	54	76	72	76	74	73	72	74	74	74	46	40	40
EguCBF D	75	**97**	68	81	67	75	72	76	73	73	72	74	73	74	47	37	40

Bolded numbers correspond to the most identical sequences (>90% identity).

### Phylogenetic relationships within CBF and DREB2 groups from *Eucalyptus*, *Arabidopsis*, *Vitis*, *Malus* and *Populus*

**Fig 3:**
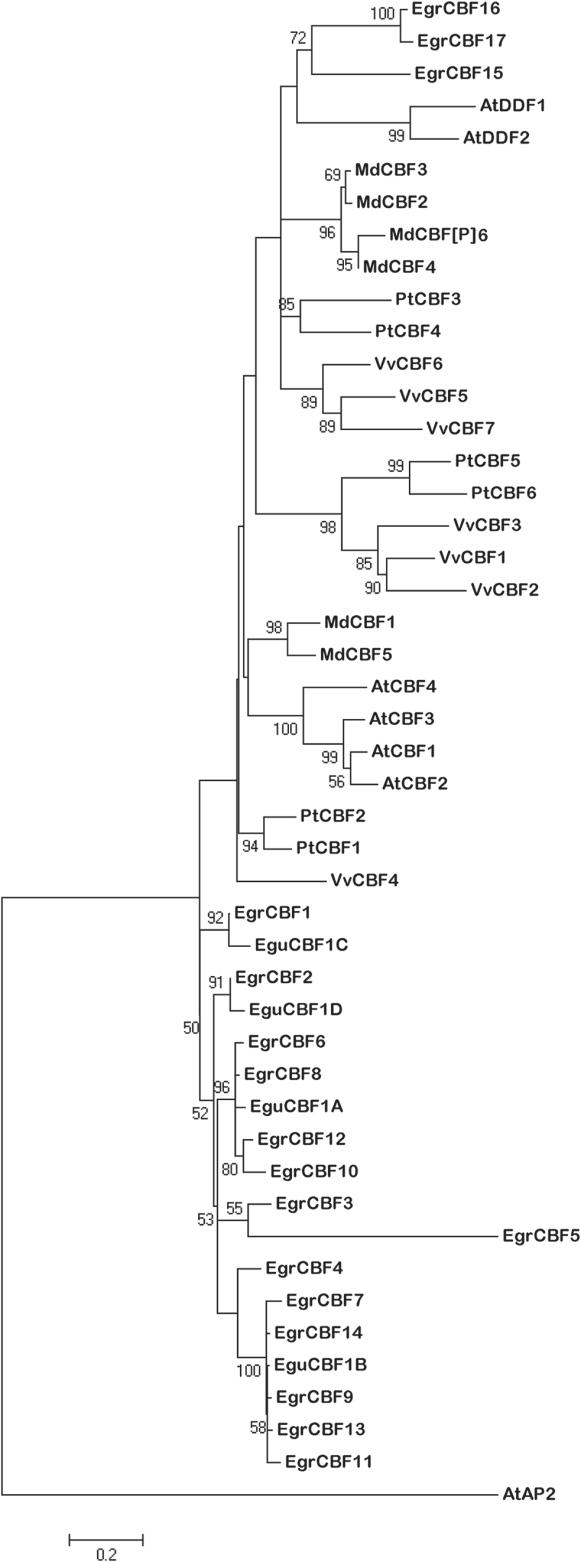
Phylogenetic analyses of CBF proteins (A1 group) from *Eucalyptus grandis*, *Eucalyptus gunnii*, *Vitis vinifera*, *Malus domestica*, *Populus trichocarpa* and *Arabidopis thaliana* by the Maximum Likelihood method using Mega 5. The tree was constructed from the sequence alignement of full-length proteins of the EgrCBF1-17 from *E*. *grandis*, Egu CBF1A-B-C-D from *E*. *gunnii*, VvCBF1-7 from *V*. *vinifera*, PtCBF1-6 from *P*. *trichocarpa*, AtCBF1-4 and AtDDF1-2 from *A*. *thaliana* and MdCBF1-6 from *Malus domestica*. AtAP2 (At5G557390.1) is a member of the AP2 subfamily used for rooting the phylogenetic tree. Low Bootstrap values (<50) were removed from the tree.

The multiple alignments of CBF protein sequences from *Arabidopsis*, *Vitis*, *Populus*, *Malus* and *Eucalyptus (E*. *grandis* and *E*. *gunnii)* were used to generate the phylogenetic tree presented in Fig 3. The CBF paralogs are distributed among either two (*Arabidopsis*, *Eucalyptus* and *Malus*) or three clades (*Vitis* and *Populus*). These clades are split into plant specific sub-clades, composed of highly-related paralogs, suggesting that gene duplications seem to have occurred in a plant species specific way. The two clades of the well-characterized *Arabidopsis* CBF proteins correspond to typical and atypical CBFs (respectively AtCBF1-4 and AtDDF1-2). The small clade (*EgrCBF*15-17) appears to be phylogenetically related to the AtDDFs. Interestingly, the main clade (*EgrCBF*1-14) corresponds to the clustered genes on scaffold 1, providing a strong evidence of repeated and recent gene duplications. This large clade might result from successive waves of duplications from one ancestor gene, the most recent leading to the two main sub-clades (CBF-A and-B like genes).

**Fig 4:**
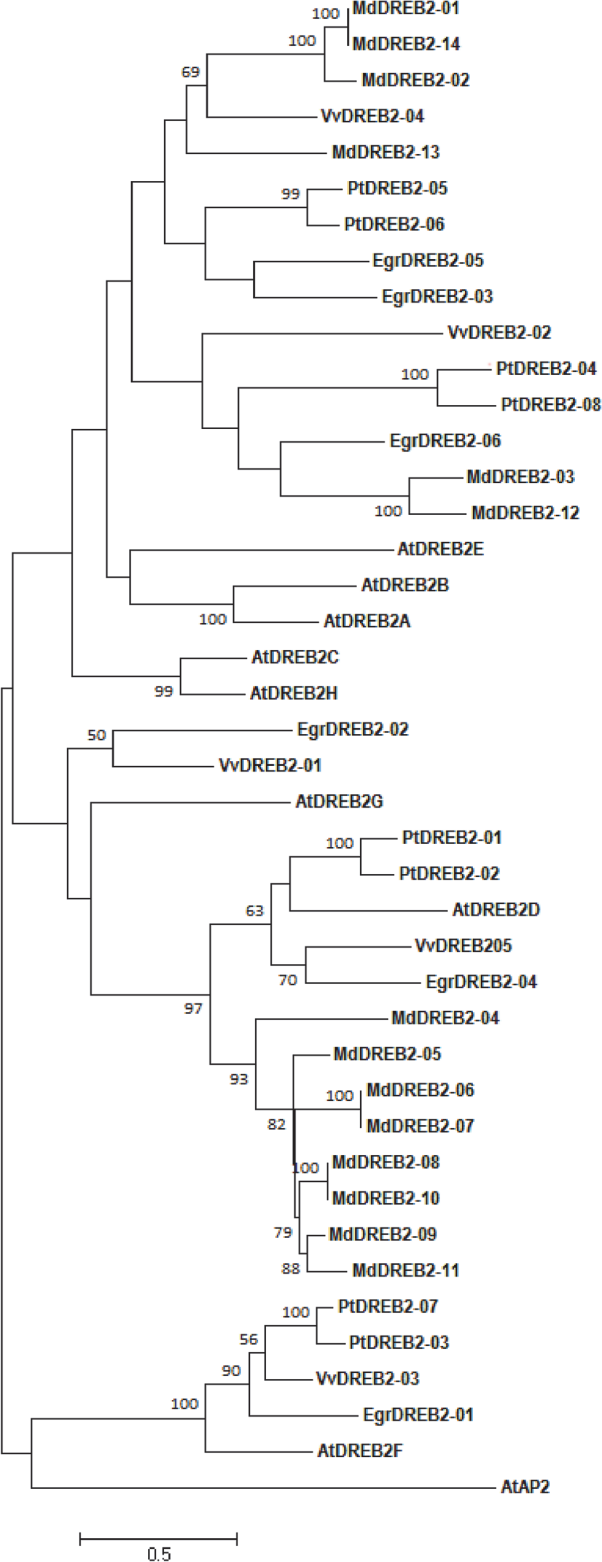
Phylogenetic analyses of DREB2 proteins (A2 group) from *Eucalyptus grandis*, *Vitis vinifera*, *Malus domestica*, *Populus trichocarpa* and *Arabidopsis thaliana* by the Maximum Likelihood method using Mega 5. The tree was constructed from the sequence alignment of full-length protein of AtDREB2-(A-H) from *A*. *thaliana*, EgrDREB2-(1–6) from *E*. *grandis*, VvDREB2-(1–5) from *V*. *vinifera*, PtDREB2-(1–8) from *P*. *trichocarpa*, MdDREB2-(1–14) from *Malus domestica*. AtAP2 (At5G557390) is a member of AP2 subfamily used for rooting the phylogenetic tree. Low Bootstrap values (<50) were removed from the tree.

The same approach with DREB2 protein sequences from *Arabidopsis*, *Vitis*, *Populus*, *Malus* and *Eucalyptus* was used for generating the DREB2 phylogenetic tree. As presented on Fig 4, the tree is divided into three main clades, each composed of paralogs from the five species under study, except for one clade with no *Malus* genes. Similarly to *Vitis* but in contrast to *Populus*, *Malus* and *Arabidopsis*, the six sequences from *Eucalyptus* are spread out over the phylogenetic tree, indicating the absence of recent duplication in this DREB group.

The phylogenetic relationships indicate that the *EgrCBF* genes result from recent, specific and repeated duplications while the *EgrDREB2* members have evolved without specific duplication from sequences prevalent already before the divergence of the species.

### Evaluation of the Egr*CBF* and Egr*DREB2* transcription in standard or stressful conditions

**Fig 5:**
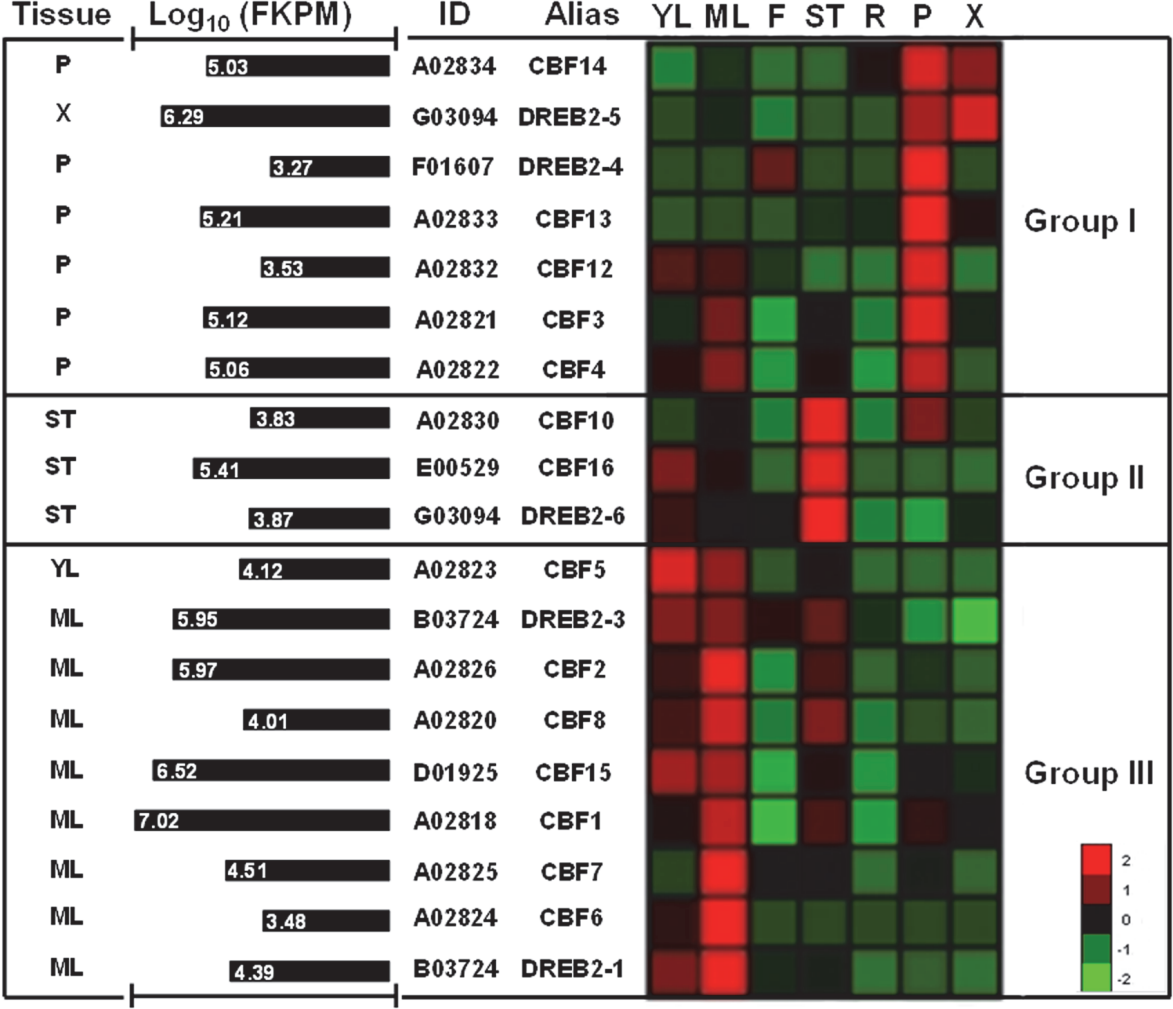
*CBF* and *DREB2* gene expression profiles from RNAseq data corresponding to various tissues from *E*. *grandis*. Raw expression data were obtained from the *Eucalyptus* genome integrative explorer (http://eucgenie.bi.up.ac.za). Expression analysis is presented for 14 *EgrCBF* and five *EgrDREB2* genes which were automatically annotated, but do not concerns the four additional CBF genes which were manually annotated in our hands. Gene name and accession numbers are mentioned respectively in Alias and ID columns. Heat map and hierarchical clustering of gene expression profiles (right panel) were performed by using expander software (ACGT, http://www.cs.tau.ac.il). For each gene, the highest absolute expression value (Log_10_ FKPM) is represented in the left panel with the indication of corresponding tissue. YL, young leaves; ML, mature leaves; F, flowers; ST, shoot tips; R, roots P, phloem; X, xylem.

The *CBF* and *DREB2* gene expression profiles were first investigated by using RNAseq data from different tissues of *E*. *grandis* plants grown in standard conditions. These data only concern five *DREB2* in addition to 14 *CBF* genes automatically annotated. As shown on Fig 5, three groups containing both *EgrCBF* and *EgrDREB2* genes can be distinguished according to their preferential expression in woody tissues (Group I), shoot tips (Group II) or leaf tissues (Group III). Each group includes both *CBF* and *DREB2* genes. Surprisingly, 6 of the 19 *CBF*/*DREB2* genes are preferentially expressed in phloem tissue in addition to *DREB2-5* which exhibits the highest expression value (Log_10_ FKPM) in xylem tissue. In the “leaf group”, the highest expression levels mainly concern mature leaves.

**Fig 6:**
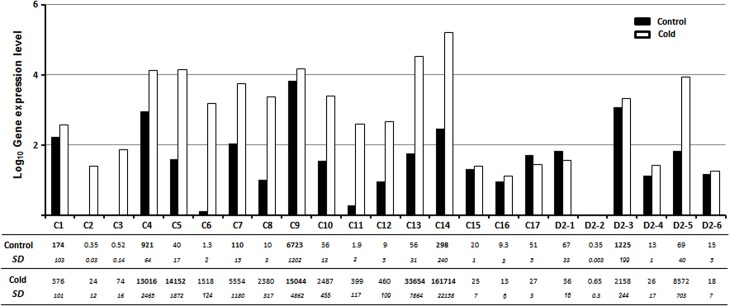
*EgrCBF* and *EgrDREB2* transcript abundance in leaves of *E*. *gunnii x E*. *dalrympleana* plants grown for 8h under 4°C cold (white) or control conditions (black). Gene expression levels of *EgrCBF* (C1 to C17) and *EgrDREB2* (D2-1 to -6) were quantified on leaves from cold-treated *Eucalyptus* plants. The experimental values from RT-qPCR (conducted on three biological replicates) were normalized using five reference genes and the mean values (n = 3) were analyzed for significance using the statistical T-test (p value < 0.05). The resulting histograms represent Log_10_ of relative transcript abundance of the *CBF* and *DREB2* in control or cold conditions. * means a significant 2-fold rate increase in gene expression (cold/control)

The *CBF* or *DREB2* transcript abundance was quantified on leaves from *E*. *gunnii x E*. *dalrympleana* cold-treated plants in comparison with standard conditions (Fig 6).This expression analysis shows that most of the *CBF* and *DREB2* genes are expressed in standard conditions. The statistical analysis indicates a significant cold induction for most of the *CBF* genes (*CBF1-14*) but only two *DREB2* genes *(DREB2-3* and*-5)*. From the 23 genes under study, *CBF14* exhibits the strongest expression under cold and *CBF9* shows the highest basal expression. Interestingly, these two genes are close orthologs of respectively *EguCBF1B* and *EguCBF1A*, which were chosen for over-expression in *E*. *urophylla x E*. *grandis* transgenic lines [24].

In addition, the responses of these genes to different stressful conditions (cold, heat or drought) were evaluated on detached leaves from *E*. *grandis*, since the use of this simplified system was validated previously [43]. In this paper, the induction of different CBF genes of cold-stressed pieces of leaves was shown to be representative of CBF expression profiles from *Eucalyptus* cold-treated plants.

**Fig 7:**
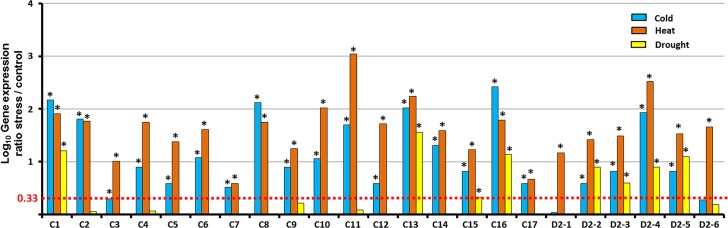
*EgrCBF* and *EgrDREB2* gene induction rates for *E*. *grandis* detached leaves incubated for 8 hours under cold (4°C, blue), heat (45°C, red) or drought (yellow), in comparison to control conditions. Transcript abundance of *EgrCBF* (C1 to C17) and *EgrDREB2* (D2-1 to -6) was quantified through RT-qPCR on detached leaves from 10 individual *E*. *grandis* plants. The experimental values were normalized using two reference genes and the mean values issued from three independent biological replicates (n = 3) were analyzed for significance using the statistical T-test (p value < 0.01). The histograms represent the relative expression values as Log_10_ of induction rates (stress/control). * means a significant 2-fold rate increase in gene expression (stress/control)

In the present paper, the gene induction rates (stress condition compared to control) are expressed (Fig 7) as the Log_10_ of the mean values (a 2-fold rate increase in gene expression corresponds to Log_10_ values > 0.33). In this experiment, all the *CBF* genes were found to be cold-induced *versus* 4/6 *DREB2* genes. In agreement with the presented data on whole plants, the overall *CBF* group exhibits a more intense cold response than the *DREB2* group. At the opposite, under drought, a higher proportion of *DREB2* genes (4/6) are up-regulated compared to the *CBF* genes (4/17), even though the applied stimulus is overall triggering a much weaker response compared to cold treatment. Finally, all the *CBF* and *DREB2* genes were found to be heat-inducible.

Overall, these analyses confirm that both *CBF* and *DREB2* groups participate in cold, drought and above all heat response in *E grandis* and *E*. *gunnii x E*. *dalrympleana*. However, CBF group would be mostly involved in cold response and DREB2 mainly in drought response. Moreover, the data evidence that, for these two species of *Eucalyptus*, regulation is not redundant within the *CBF/DREB2* groups since each gene exhibits a distinct pattern of response in different tissues and stressful conditions.

## Discussion

A comprehensive search for the *Eucalyptus* AP2/ERF transcription factors led to the identification of 202 genes which is similar to *Populus* and ranges in between *Vitis* (147 genes) and *Zea mays* (292 members) [51]. A previous comparison of the *AP2/ERF* gene family size between eudicots, monocots and chlorophyta, have already shown that this size radiates along with land conquest [52]. Actually, most of the observed expansions in AP2/ERF families in plants seem to be associated to recent genome duplications (*Eucalyptus*, *Populus and Malus) or triplications (Brassica rapa*) [53], [54], [55], [23]. The structure and phylogeny of the AP2/ERF family are similar for the species under study. The presence of the same subfamilies composed of the same groups suggests that the appearance of the AP2/ERF structure predates the divergence of dicotyledonous species [28, 47]. Compared to Arabidopsis, the tree species under study are characterized by a much larger ERF subfamily beside a similar size for DREB subfamily. Current knowledge states that DREB factors activate genes involved in cold and water deficit response, while ERF factors directly regulate pathogenesis-related (PR) gene expression but some *ERF* and *DREB* genes were shown to be activated by both biotic and abiotic stimuli [56]. Actually, most of these ERF in *Eucalyptus* are B3 factors. More than other ones, these proteins are often associated to the response to both biotic and abiotic stresses, especially through the binding to CE1 element [57],[58], [59].

With regard to the *Eucalyptus DREB* subfamily, the obvious feature lies in the presence of numerous *EgrCBF* genes together with a moderate number of *EgrDREB*2 genes. The phylogenetic analysis suggests that the *DREB*2 forms existed prior to the plant species divergence, whereas the *CBF* may have evolved more recently and independently in each plant species. Up to now, the number of *CBF* genes in dicotyledonous plants is often around six, except for *Chrysanthemum* [60] and *Medicago truncatula* [61]. In the first case, the 13 *CBF* isolated genes are likely related to the hexaploid *Chrysanthemum* genome; 17 *CBF* genes have been detected in *M*. *truncatula* genome which has experienced high rates of local gene duplications.

The occurrence of clustered *CBF* genes is a quite common feature for several plant species such as *Arabidopsis*, wheat and rice [9, 25, 62], but the number of *CBF* duplication events is much higher in *Medicago* and *Eucalyptus*. Duplicated genes can be conserved and associated with sub-functionalization and/or neo-functionalization [63] or become pseudogenes. The intriguing high number of *EgrCBF* genes may give rise to doubts as to their transcription. Given that all the *EgrCBF*s are expressed and that only one is predicted not to be translated, it then raises questions about the role of these very similar multiple copies in *Eucalyptus*. Are they required to ensure an essential function or is there redundancy in their function? Knox and coworkers showed that the presence of two extra-*CBF* gene copies in a barley genotype contributes to its higher freezing tolerance [25], suggesting that amplification of genomic regions encompassing *CBF*s would be a mean to increase *CBF* transcription rate and provide a selective advantage during winter. Also the *CBF* family expansion in the *Pooideae* during the Eocene-Oligocene climate cooling was strongly suggested to play a role in the adaptation to a cool climate [64]. Considering its tropical and subtropical origin and ever-growing habit, *E*. *grandis* is reported to exhibit a surprisingly high level of frost tolerance as a result of a very rapid hardening capacity [65]. *Eucalyptus* genus is also known as heat-tolerant [66]. The *CBF* could, at least in part, participate in the control of the *Eucalyptus* cold acclimation since over-expression of an *E*. *gunnii CBF* ortholog in *Eucalyptus* was found to increase cold tolerance [24]. Interestingly, the protein corresponding to this transgene is very similar (98–99%) to EgrCBF13 and 14 which were observed as the most expressed in the present paper, such validating their functionality. Therefore, duplication and retention of the *CBF* genes, most cold- and heat-responsive may contribute to a massive activation of target genes (such as dehydrin) as previously observed during *Eucalyptus* cold acclimation [67].

On the other hand, the different copies may be not equivalent for regulation or function, at least at the subgroup level. In *Arabidopsis*, the divergence of the *CBF*4 gene from the *CBF*1-3 tandem proved not to be due to selection of entirely new functions, but to diversification of promoter regulatory elements responding to different environmental signals [68, 69]. In *E*. *gunnii*, previous studies have shown a complementary regulation of the four *CBF* genes under various cold conditions [33]. The present study clearly shows that the expression of the 17 *EgrCBF* genes is differential according to the plant tissue and the applied stress. Not only these genes are not specific to one stress but also they are not equivalent for regulation. Therefore the high number of *CBF* copies may provide both efficiency and plasticity to the *Eucalyptus* response to environmental stresses.

The unusual *CBF* over-representation, in contrast to the moderate number of *DREB*2 genes in *Eucalyptus* also raises the question about the adaptation of this species to cold, heat or drought. It was originally thought that *CBF* genes are the main regulators of cold response when *DREB2* group is rather involved in osmotic response, but accumulation of genomic data on different species have shown some conflicts with respect to this classical model [7]. The present study on *Eucalyptus* species shows that both groups are regulated by both stimuli, but *CBF* are more cold-sensitive than *DREB2* genes which look more involved in drought response. The regulation of *CBF* genes by various dehydrative stresses was previously shown in *E*. *gunnii* [33], *Vitis* [70], *Malus* [21], *Prunus* [22] and *Populus* [50]. In *Arabidopsis*, the *CBF* group includes the strictly cold-induced genes (*CBF1-3*) together with the drought- and salt-responsive genes (*CBF4*, *DDF1* and *DDF2*) [71]. Conversely, cold-responsive *DREB2* genes have been reported for some grass species [56, 72] *Arabidopsis* [58], *Malus* [6] or *Populus* [50]. Therefore, the numerous *CBF* and *DREB2* genes may together be involved in the control of temperature stress in *Eucalyptus*. One obvious characteristic of *CBF/DREB2* genes with regard to transcriptional regulation in *Eucalyptus* lies in the strong response to heat which concerns all the 23 members. This feature does not look so common since for example the recent publication [73] on heat transcriptome in poplar states only 19 up-regulated *DREB* genes *versus* 23 down-regulated for the six groups (A1-A6 containing 60 genes).

From our results, one may therefore hypothesize that, in *Eucalyptus*, the large *CBF* group would complete the limited number of *DREB2* genes, for regulating multiple abiotic stress responses. This does not seem so surprising because expansion of *Eucalyptus* into Australia occurred during the late Miocene when the climate became colder and drier [74]. This means that this tree was submitted to a double selection pressure. As regards the cold response, the higher number of *CBF* genes in *Eucalyptus* compared to other trees highlights the very distinct functional strategies to face winter mainly based on frost tolerance for *Eucalyptus versus* frost avoidance for *Populus* or *Malus*. In addition, all the *CBF* and *DREB*2 genes exhibit a significant induction by heat stress. It is now commonly accepted that *Eucalyptus* became the dominant sclerophyll forest in Australia, about 140 000 years ago, when Aboriginal people arrived because of “fire dick” agriculture [75]. It is proposed that *Eucalyptus* plants were merely pre-adapted for fire survival and expansion [66]. The presence of a large number of heat-responsive *CBFs* might be a piece of this pre-adaptation by controlling proteins involved in heat protection, like HSP. In conclusion, *CBF* and *DREB*2 may be key players of cross-talks between cold, heat and drought responses which appear to be strong in *Eucalyptus* both for the regulatory pathways and the resulting adaptive mechanisms. The unusual expansion of the *EgrCBF* group, together with a differential gene regulation could be a way of counterbalancing the permanent exposure to a changing environment and may contribute to a wider response to a range of environmental changes including at least heat shock.

In conclusion, this investigation of the AP2/ERF family in *Eucalyptus* with regard to other woody species reveals some common features, such as family size and global structure. As more and more reported for plants, the stress-related groups *CBF* and *DREB2* prove differentially but not specifically regulated by various abiotic stresses, *CBF* members participating mainly in the cold response and *DREB*2 genes in the drought response. More surprising with regard to other species is the strong response to heat stress for all the *CBF* and *DREB2* members.

Above all, *Eucalyptus* genome is marked out by a dramatic over-representation of *CBF* genes, which does not appear like a woody plant feature. This result gives evidence of intense and recent duplication events and suggests their importance in the adaptation of this ever-growing tree to cold, drought and above all heat. Overall, this result strongly supports the hypothesis of Myburg and coworkers [3] that the intense genome-wide duplications in *Eucalyptus* (the highest reported to date) may be important for adaptive evolution of this tree in dynamically changing environments.

Finally, these data make now possible to envisage a functional characterization including spatiotemporal expression studies for identifying the most relevant candidate genes (*CBF* and *DREB2*) towards improving stress tolerance (cold, drought or combined abiotic stresses).

## Supporting Information

S1 Fig Phylogenetic tree of the DREB subfamily from *A*. *thaliana* and *E*. *grandis*. The tree was generated using Mega 5 program by the Maximum Likelihood method. Bootstrap values are indicated at each branch. For the *E*. *grandis* DREB subfamily, the relation-ship between Phytozome gene IDs and the names used in this paper is indicated in S2 Table.
(TIF)

S2 Fig Phylogenetic tree of the ERF subfamily from *A*. *thaliana* and *E*. *grandis*. The tree was generated using Mega 5 program by the Maximum Likelihood method. Bootstrap values are indicated at each branch. For the *E*. *grandis* ERF subfamily, the relation-ship between Phytozome gene IDs and the names used in this paper is indicated in S2 Table.
(TIF)

S3 Fig Phylogenetic tree of the RAV subfamily from *A*. *thaliana* and *E*. *grandis*. The tree was generated using Mega 5 program by the Maximum Likelihood method. Bootstrap values are indicated at each branch. For the *E*. *grandis* RAV subfamily, the relation-ship between Phytozome gene IDs and the names used in this paper is indicated in S2 Table.
(TIF)

S4 Fig Phylogenetic tree of the AP2 subfamily from *A*. *thaliana* and *E*. *grandis*. The tree was generated using Mega 5 program by Maximum Likelihood method. Bootstrap values are indicated at each branch. For the *E*. *grandis* AP2 subfamily, subfamily, the relation-ship between Phytozome gene IDs and the names used in this paper is indicated in S2 Table.
(TIF)

S1 Table List of oligonucleotide sequences used in RT-qPCR reactions; the Tm and the amplicon size (bp) are provided. (DOC)

S2 Table Correspondence between AP2/ERF gene IDs from phytozome and names used in this paper. “Eucagen name” associates the subfamily name with the relative position on the scaffolds (1 to 11). “Generic name” given to EgrCBF(1–17) and EgrDREB2-(1–6) from literature uses and genome location.
(DOC)
