# Supplementary figures and images for: Genome-Wide Analysis of the AP2/ERF Family in Eucalyptus grandis: An Intriguing Over-Representation of Stress-Responsive DREB1/CBF Genes
Source: PLoS One. 2015 Apr 7;10(4):e0121041. doi: 10.1371/journal.pone.0121041 (PMC4388522; doi:10.1371/journal.pone.0121041)

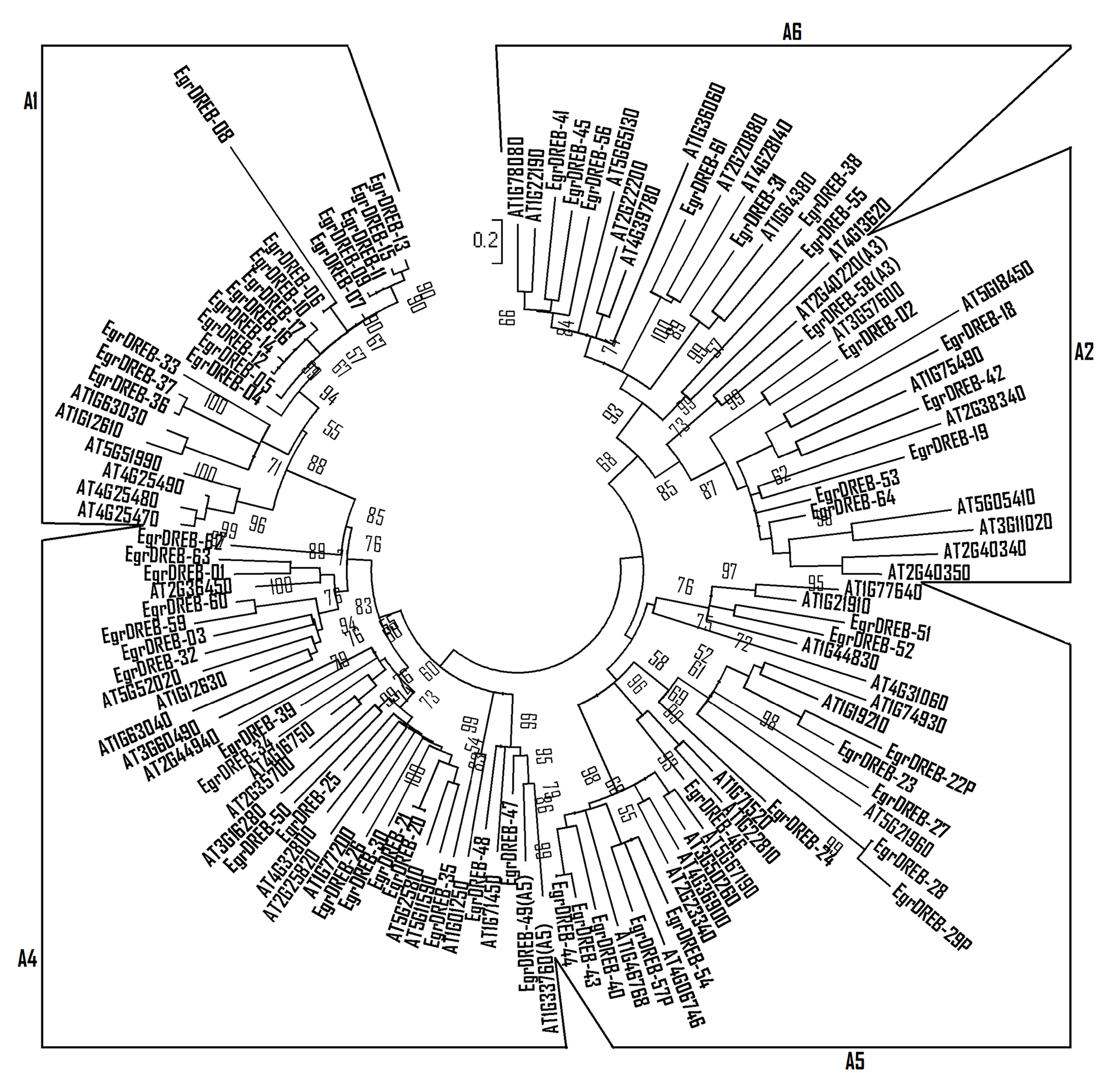

Supplement: S1 Fig — The tree was generated using Mega 5 program by the Maximum Likelihood method. Bootstrap values are indicated at each branch. For the E. grandis DREB subfamily, the relation-ship between Phytozome gene IDs and the names used in this paper is indicated in S2 Table. (TIF) [file pone.0121041.s001.tif]

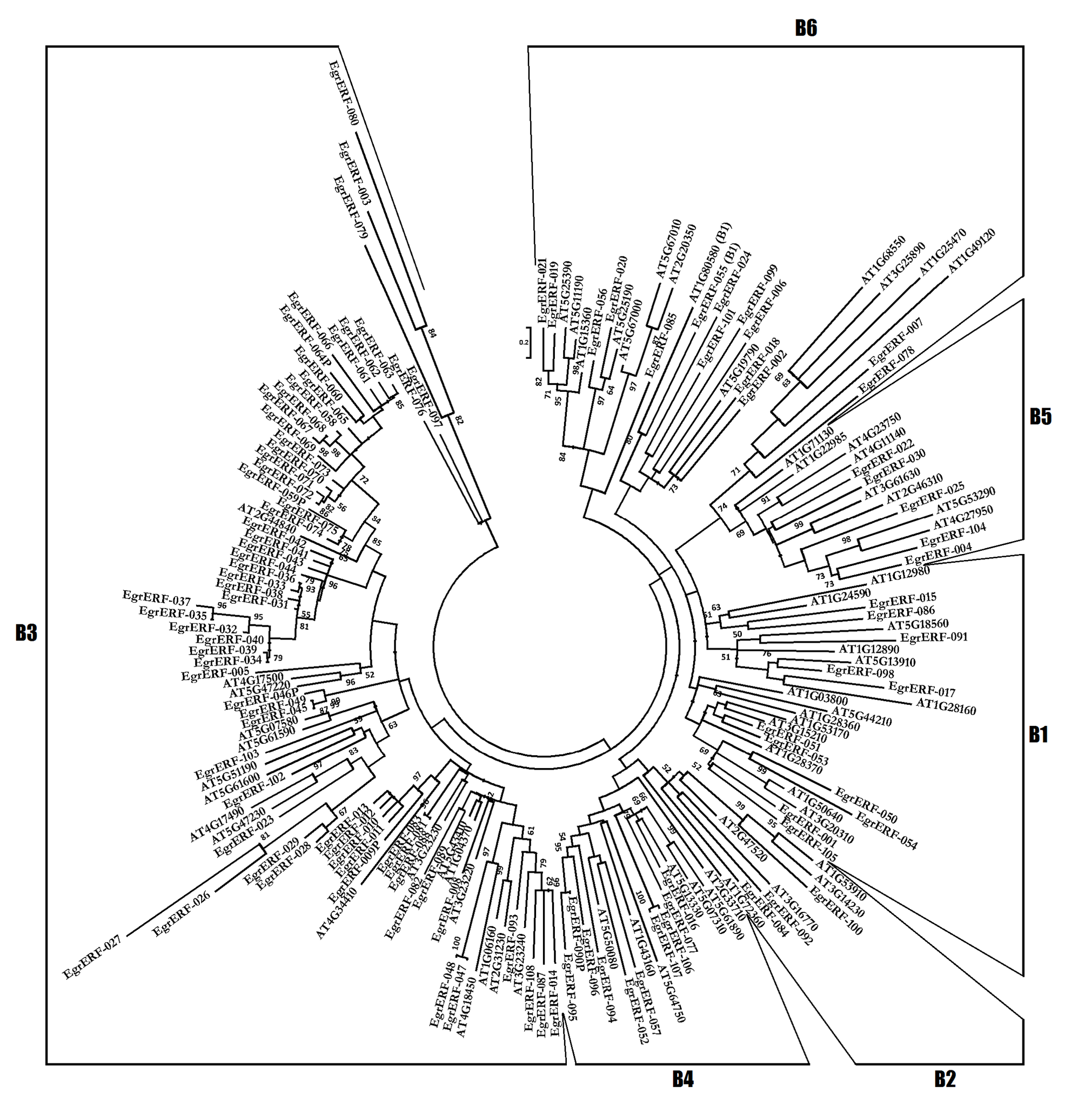

Supplement: S2 Fig — The tree was generated using Mega 5 program by the Maximum Likelihood method. Bootstrap values are indicated at each branch. For the E. grandis ERF subfamily, the relation-ship between Phytozome gene IDs and the names used in this paper is indicated in S2 Table. (TIF) [file pone.0121041.s002.tif]

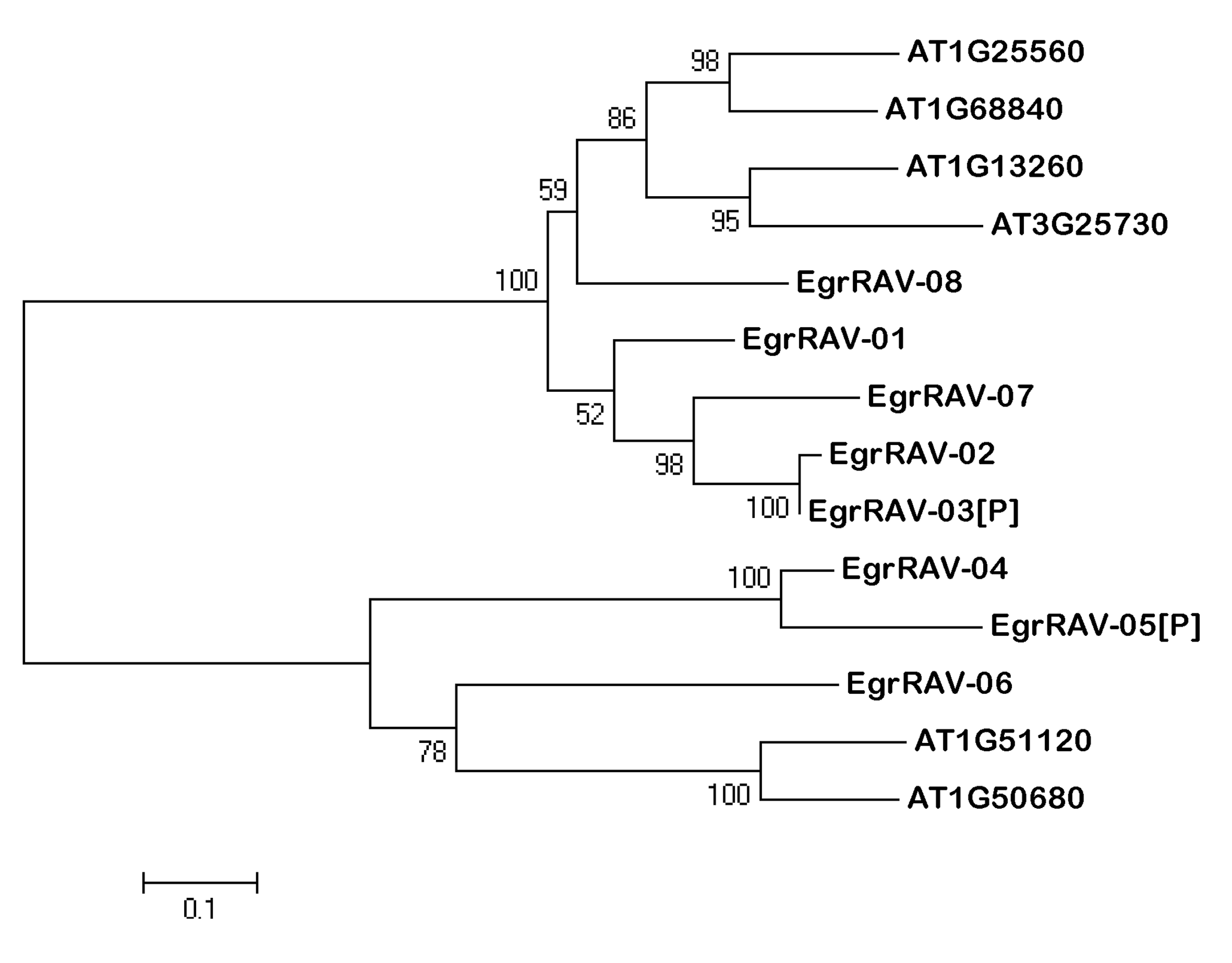

Supplement: S3 Fig — The tree was generated using Mega 5 program by the Maximum Likelihood method. Bootstrap values are indicated at each branch. For the E. grandis RAV subfamily, the relation-ship between Phytozome gene IDs and the names used in this paper is indicated in S2 Table. (TIF) [file pone.0121041.s003.tif]

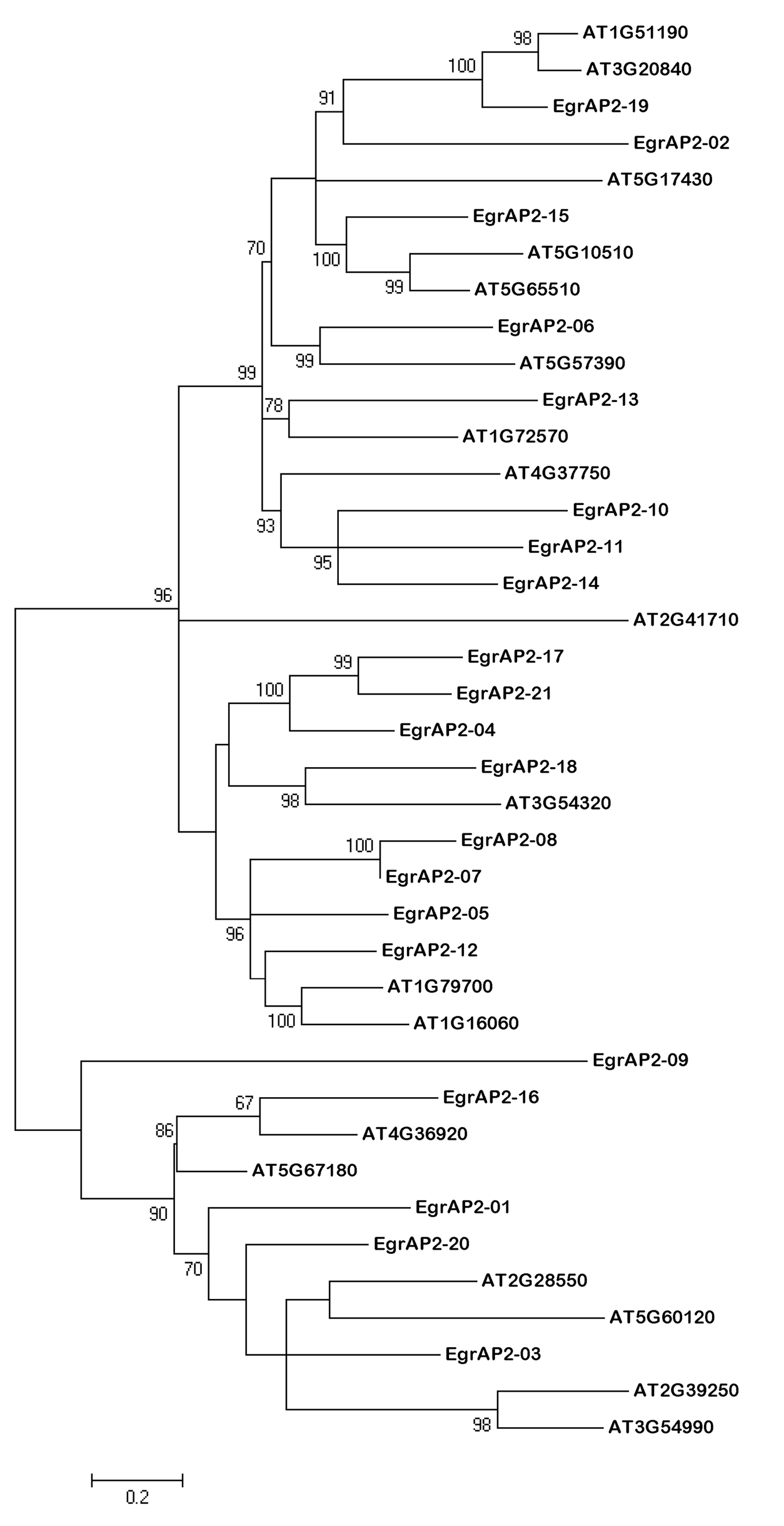

Supplement: S4 Fig — The tree was generated using Mega 5 program by Maximum Likelihood method. Bootstrap values are indicated at each branch. For the E. grandis AP2 subfamily, subfamily, the relation-ship between Phytozome gene IDs and the names used in this paper is indicated in S2 Table. (TIF) [file pone.0121041.s004.tif]
