# Supplementary material for: Genome-Wide Analysis of the AP2/ERF Family in Eucalyptus grandis: An Intriguing Over-Representation of Stress-Responsive DREB1/CBF Genes
Source: PLoS One. 2015 Apr 7;10(4):e0121041. doi: 10.1371/journal.pone.0121041 (PMC4388522; doi:10.1371/journal.pone.0121041)
Supplement: S2 Table — “Eucagen name” associates the subfamily name with the relative position on the scaffolds (1 to 11). “Generic name” given to EgrCBF(1–17) and EgrDREB2-(1–6) from literature uses and genome location. (DOC) [file pone.0121041.s006.doc]

**Table S2: Correspondence between AP2/ERF gene IDs from phytozome and names used in this paper.**

| **Eucagen name** | **Generic name** | **Old Phytozome name** | **New Phytozome name** | **Genome location** | **Scaffold** |
| --- | --- | --- | --- | --- | --- |
| EgrAP2-01 |  | Egrandis_v1_0.025322m | Eucgr.A01182.1 | 18327857..18330514 | 1 |
| EgrAP2-02 |  | Egrandis_v1_0.004950m | Eucgr.B01460.1 | 23959204..23962641 | 2 |
| EgrAP2-03 |  | Egrandis_v1_0.012016m | Eucgr.B02453.1 | 46667137..46670344 | 2 |
| EgrAP2-04 |  | Egrandis_v1_0.050917m | Eucgr.B03412.1 | 58906723..58908760 | 2 |
| EgrAP2-05 |  | Egrandis_v1_0.042166m | Eucgr.C00238.1 | 4981692..4984577 | 3 |
| EgrAP2-06 |  | Egrandis_v1_0.008536m | Eucgr.C02333.1 | 43512245..43515307 | 3 |
| EgrAP2-07 |  | Egrandis_v1_0.049039m | Eucgr.C02519.1 | 48063883..48065768 | 3 |
| EgrAP2-08 |  | Egrandis_v1_0.045511m | Eucgr.C02520.1 | 48072094..48074796 | 3 |
| EgrAP2-09 |  | nd | nd | 3509070..3509792 | 5 |
| EgrAP2-10 |  | Egrandis_v1_0.008385m | Eucgr.F00098.1 | 1874570..1877293 | 6 |
| EgrAP2-11 |  | Egrandis_v1_0.005293m | Eucgr.F02223.1 | 30145920..30149339 | 6 |
| EgrAP2-12 |  | Egrandis_v1_0.017864m | Eucgr.F03987.1 | 48122085..48125896 | 6 |
| EgrAP2-13 |  | Egrandis_v1_0.006443m | Eucgr.F04421.1 | 53193313..53197116 | 6 |
| EgrAP2-14 |  | Egrandis_v1_0.011721m | Eucgr.H02335.1 | 31368271..31371124 | 8 |
| EgrAP2-15 |  | Egrandis_v1_0.039221m | Eucgr.I00564.1 | 11600673..11605220 | 9 |
| EgrAP2-16 |  | Egrandis_v1_0.007978m | Eucgr.I00892.1 | 18319013..18322320 | 9 |
| EgrAP2-17 |  | Egrandis_v1_0.039369m | Eucgr.I01921.1 | 29141662..29143666 | 9 |
| EgrAP2-18 |  | Egrandis_v1_0.014231m | Eucgr.J00316.1 | 3179352..3183688 | 10 |
| EgrAP2-19 |  | Egrandis_v1_0.054287m | Eucgr.J00792.1 | 8408831..8411806 | 10 |
| EgrAP2-20 |  | Egrandis_v1_0.010819m | Eucgr.J02113.1 | 27123919..27127106 | 10 |
| EgrAP2-21 |  | Egrandis_v1_0.053635m | Eucgr.J02131.1 | 27270076..27271944 | 10 |
|  |  |  |  |  |  |
| EgrDREB-01 |  | Egrandis_v1_0.031215m | Eucgr.A01537.1 | 24002524..24003087 | 1 |
| EgrDREB-02 | EgrDREB2-1 | Egrandis_v1_0.021692m | Eucgr.A02390.1 | 34657029..34657901 | 1 |
| EgrDREB-03 |  | Egrandis_v1_0.024019m | Eucgr.A02817.1 | 38645758..38646519 | 1 |
| EgrDREB-04 | EgrCBF1 | Egrandis_v1_0.025448m | Eucgr.A02818.1 | 38661144..38661833 | 1 |
| EgrDREB-05 | EgrCBF2 | Egrandis_v1_0.044344m | Eucgr.A02820.1 | 38683953..38684543 | 1 |
| EgrDREB-06 | EgrCBF3 | Egrandis_v1_0.042687m | Eucgr.A02821.1 | 38689094..38689595 | 1 |
| EgrDREB-07 | EgrCBF4 | Egrandis_v1_0.025684m | Eucgr.A02822.1 | 38695421..38695993 | 1 |
| EgrDREB-08 | EgrCBF5 | Egrandis_v1_0.048068m | Eucgr.A02823.1 | 38698022..38698666 | 1 |
| EgrDREB-09 | EgrCBF6 | Egrandis_v1_0.051954m | Eucgr.A02824.1 | 38703149..38703811 | 1 |
| EgrDREB-10 | EgrCBF7 | Egrandis_v1_0.050421m | Eucgr.A02825.1 | 38707931..38708605 | 1 |
| EgrDREB-11 | EgrCBF8 | Egrandis_v1_0.042378m | Eucgr.A02826.1 | 38716577..38717239 | 1 |
| EgrDREB-12 | EgrCBF9 | Egrandis_v1_0.045584m | Eucgr.A02827.1 | 38721707..38722381 | 1 |
| EgrDREB-13 | EgrCBF10 | Egrandis_v1_0.026041m | Eucgr.A02830.1 | 38740722..38741384 | 1 |
| EgrDREB-14 | EgrCBF11 | Egrandis_v1_0.053880m | Eucgr.A02831.1 | 38756517..38757191 | 1 |
| EgrDREB-15 | EgrCBF12 | Egrandis_v1_0.040541m | Eucgr.A02832.1 | 38761183..38761845 | 1 |
| EgrDREB-16 | EgrCBF13 | Egrandis_v1_0.028907m | Eucgr.A02833.1 | 38766700..38767468 | 1 |
| EgrDREB-17 | EgrCBF14 | Egrandis_v1_0.025814m | Eucgr.A02834.1 | 38777037..38777711 | 1 |
| EgrDREB-18 | EgrDREB2-2 | Egrandis_v1_0.051078m | Eucgr.B02161.1 | 42814072..42814785 | 2 |
| EgrDREB-19 [P] | EgrDREB2-3 | Egrandis_v1_0.014873m | Eucgr.B03724.1 | 61583661..61584038 | 2 |
| EgrDREB-20 |  | Egrandis_v1_0.024797m | Eucgr.C00546.1 | 9732214..9732933 | 3 |
| EgrDREB-21 |  | Egrandis_v1_0.029313m | Eucgr.C00590.1 | 11211766..11212485 | 3 |
| EgrDREB-22 [P] |  | nd | nd | 12861672..12986569 | 3 |
| EgrDREB-23 |  | Egrandis_v1_0.024729m | Eucgr.C00780.1 | 12995821..12996543 | 3 |
| EgrDREB-24 |  | Egrandis_v1_0.028677m | Eucgr.C00781.1 | 12997434..12997967 | 3 |
| EgrDREB-25 |  | nd | nd | 16042747..16043586 | 3 |
| EgrDREB-26 |  | Egrandis_v1_0.023726m | Eucgr.C02928.1 | 54970759..54971677 | 3 |
| EgrDREB-27 |  | Egrandis_v1_0.026845m | Eucgr.C03175.1 | 60262835..60263458 | 3 |
| EgrDREB-28 |  | Egrandis_v1_0.027893m | Eucgr.C03297.1 | 62492800..62493426 | 3 |
| EgrDREB-29 [P] |  | nd | nd | 62496239.62496838 | 3 |
| EgrDREB-30 |  | Egrandis_v1_0.025181m | Eucgr.C03785.1 | 71249324..71250025 | 3 |
| EgrDREB-31 |  | Egrandis_v1_0.041217m | Eucgr.D01731.1 | 31330435..31331340 | 4 |
| EgrDREB-32 |  | Egrandis_v1_0.023349m | Eucgr.D01924.1 | 33211928..33212722 | 4 |
| EgrDREB-33 | EgrCBF15 | Egrandis_v1_0.024787m | Eucgr.D01925.1 | 33231380..33232102 | 4 |
| EgrDREB-34 |  | Egrandis_v1_0.037887m | Eucgr.D02102.1 | 35124249..35124926 | 4 |
| EgrDREB-35 |  | Egrandis_v1_0.040558m | Eucgr.E00046.1 | 449330..449875 | 5 |
| EgrDREB-36 | EgrCBF16 | Egrandis_v1_0.021707m | Eucgr.E00529.1 | 5019457..5020326 | 5 |
| EgrDREB-37 | EgrCBF17 | Egrandis_v1_0.045089m | Eucgr.E00530.1 | 5046264..5047124 | 5 |
| EgrDREB-38 |  | Egrandis_v1_0.019614m | Eucgr.E00857.1 | 9010804..9011772 | 5 |
| EgrDREB-39 |  | Egrandis_v1_0.050093m | Eucgr.E01593.1 | 19357525..19358394 | 5 |
| EgrDREB-40 |  | Egrandis_v1_0.029340m | Eucgr.F00192.1 | 2935908..2936408 | 6 |
| EgrDREB-41 |  | Egrandis_v1_0.017137m | Eucgr.F00497.1 | 6306729..6307814 | 6 |
| EgrDREB-42 | EgrDREB2-4 | Egrandis_v1_0.050374m | Eucgr.F01607.1 | 20368999..20369706 | 6 |
| EgrDREB-43 |  | Egrandis_v1_0.041817m | Eucgr.F02317.1 | 31616037..31616507 | 6 |
| EgrDREB-44 |  | Egrandis_v1_0.050025m | Eucgr.F02319.1 | 31636048..31636491 | 6 |
| EgrDREB-45 |  | Egrandis_v1_0.018748m | Eucgr.F02440.1 | 34000315..34001322 | 6 |
| EgrDREB-46 |  | Egrandis_v1_0.029984m | Eucgr.F02967.1 | 39331097..39331564 | 6 |
| EgrDREB-47 |  | Egrandis_v1_0.050609m | Eucgr.F02974.1 | 39373057..39373593 | 6 |
| EgrDREB-48 |  | Egrandis_v1_0.042888m | Eucgr.F02975.1 | 39387000..39387563 | 6 |
| EgrDREB-49 |  | Egrandis_v1_0.053262m | Eucgr.F02976.1 | 39393340..39393906 | 6 |
| EgrDREB-50 |  | Egrandis_v1_0.022607m | Eucgr.F03062.1 | 40049653..40050483 | 6 |
| EgrDREB-51 |  | Egrandis_v1_0.023644m | Eucgr.F03772.1 | 45911857..45912636 | 6 |
| EgrDREB-52 |  | Egrandis_v1_0.027741m | Eucgr.F04365.1 | 52539448..52540101 | 6 |
| EgrDREB-53 | EgrDREB2-5 | Egrandis_v1_0.024022m | Eucgr.G03094.1 | 49491517..49492311 | 7 |
| EgrDREB-54 |  | Egrandis_v1_0.029951m | Eucgr.H02094.1 | 27302029..27302499 | 8 |
| EgrDREB-55 |  | Egrandis_v1_0.014114m | Eucgr.H02782.1 | 39949478..39950725 | 8 |
| EgrDREB-56 |  | Egrandis_v1_0.049649m | Eucgr.I00422 | 8173138..8174187 | 9 |
| EgrDREB-57 [P] |  | nd | nd | 18261224..18261586 | 9 |
| EgrDREB-58 |  | Egrandis_v1_0.047916m | Eucgr.J00187.1 | 1949518..1950829 | 10 |
| EgrDREB-59 |  | Egrandis_v1_0.051885m | Eucgr.K00217.1 | 2651313..2652050 | 11 |
| EgrDREB-60 |  | Egrandis_v1_0.055153m | Eucgr.K00220.1 | 2742301..2742825 | 11 |
| EgrDREB-61 |  | Egrandis_v1_0.011144m | Eucgr.K00961.1 | 11655257..11656687 | 11 |
| EgrDREB-62 |  | Egrandis_v1_0.048153m | Eucgr.K02069.1 | 27110275..27110820 | 11 |
| EgrDREB-63 |  | Egrandis_v1_0.048281m | Eucgr.K02071.1 | 27183013..27183702 | 11 |
| EgrDREB-64 |  | Egrandis_v1_0.049567m | Eucgr.K02952.1 | 37622356..37623026 | 11 |
|  |  |  |  |  |  |
| EgrERF-001 |  | Egrandis_v1_0.052356m | Eucgr.A01091.1 | 16598553..16599230 | 1 |
| EgrERF-002 |  | Egrandis_v1_0.039599m | Eucgr.A02109.1 | 31807050..31807937 | 1 |
| EgrERF-003 |  | Egrandis_v1_0.013549m | Eucgr.A02341.1 | 34195357..34199128 | 1 |
| EgrERF-004 |  | Egrandis_v1_0.017345m | Eucgr.A02669.1 | 37291594..37292670 | 1 |
| EgrERF-005 |  | Egrandis_v1_0.045365m | Eucgr.A02924.1 | 39680687..39681532 | 1 |
| EgrERF-006 |  | Egrandis_v1_0.026873m | Eucgr.B00666.1 | 8432684..8433430 | 2 |
| EgrERF-007 |  | Egrandis_v1_0.018567m | Eucgr.B00720.1 | 8975856..8976872 | 2 |
| EgrERF-008 |  | Egrandis_v1_0.044458m | Eucgr.B01537.1 | 25639355..25639801 | 2 |
| EgrERF-009 [P] |  | Egrandis_v1_0.054396m | Eucgr.B01538.1 | 25641315..25641177 | 2 |
| EgrERF-010 |  | Egrandis_v1_0.040030m | Eucgr.B01539.1 | 25654429..25654929 | 2 |
| EgrERF-011 |  | Egrandis_v1_0.038908m | Eucgr.B01540.1 | 25667405..25667890 | 2 |
| EgrERF-012 |  | Egrandis_v1_0.049829m | Eucgr.B01541.1 | 25676489..25676974 | 2 |
| EgrERF-013 |  | Egrandis_v1_0.053506m | Eucgr.B01542.1 | 25688512..25688994 | 2 |
| EgrERF-014 |  | Egrandis_v1_0.042158m | Eucgr.B01544.1 | 25737023..25737709 | 2 |
| EgrERF-015 |  | Egrandis_v1_0.055070m | Eucgr.B03281.1 | 57719205..57720428 | 2 |
| EgrERF-016 |  | Egrandis_v1_0.024837m | Eucgr.B03559.1 | 60204008..60206584 | 2 |
| EgrERF-017 |  | Egrandis_v1_0.041191m | Eucgr.B03565.1 | 60250395..60251294 | 2 |
| EgrERF-018 |  | Egrandis_v1_0.021710m | Eucgr.B03605.1 | 60524172..60525041 | 2 |
| EgrERF-019 |  | Egrandis_v1_0.025520m | Eucgr.C01178.1 | 18251959..18252990 | 3 |
| EgrERF-020 |  | Egrandis_v1_0.028133m | Eucgr.C02719.1 | 52221167..52221812 | 3 |
| EgrERF-021 |  | Egrandis_v1_0.026700m | Eucgr.C04221.1 | 77338705..77339426 | 3 |
| EgrERF-022 |  | Egrandis_v1_0.047890m | Eucgr.D01775.1 | 31731790..31732749 | 4 |
| EgrERF-023 |  | Egrandis_v1_0.054175m | Eucgr.D02111.1 | 35241210..35242256 | 4 |
| EgrERF-024 |  | Egrandis_v1_0.040767m | Eucgr.D02238.1 | 36504755..36505684 | 4 |
| EgrERF-025 |  | Egrandis_v1_0.042138m | Eucgr.D02344.1 | 37316881..37317846 | 4 |
| EgrERF-026 |  | nd | nd | 22653..23138 | 5 |
| EgrERF-027 |  | nd | nd | 3532559..3533953 | 5 |
| EgrERF-028 |  | Egrandis_v1_0.045634m | Eucgr.E00377.1 | 3561949..3562935 | 5 |
| EgrERF-029 |  | Egrandis_v1_0.019065m | Eucgr.E00378.1 | 3567402..3568394 | 5 |
| EgrERF-030 |  | Egrandis_v1_0.019390m | Eucgr.E00834.1 | 8812221..8813198 | 5 |
| EgrERF-031 |  | Egrandis_v1_0.042412m | Eucgr.E01552.1 | 18815694..18816660 | 5 |
| EgrERF-032 |  | Egrandis_v1_0.048717m | Eucgr.E01554.1 | 18818941..18819489 | 5 |
| EgrERF-033 |  | Egrandis_v1_0.042313m | Eucgr.E01555.1 | 18825475..18826061 | 5 |
| EgrERF-034 |  | Egrandis_v1_0.046701m | Eucgr.E01556.1 | 18836113..18836823 | 5 |
| EgrERF-035 |  | Egrandis_v1_0.038821m | Eucgr.E01557.1 | 18870857..18871552 | 5 |
| EgrERF-036 |  | Egrandis_v1_0.041591m | Eucgr.E01558.1 | 18874668..18875252 | 5 |
| EgrERF-037 |  | Egrandis_v1_0.040599m | Eucgr.E01559.1 | 18900721..18901245 | 5 |
| EgrERF-038 |  | Egrandis_v1_0.049543m | Eucgr.E01560.1 | 18904326..18904913 | 5 |
| EgrERF-039 |  | Egrandis_v1_0.053720m | Eucgr.E01561.1 | 18932811..18936085 | 5 |
| EgrERF-040 |  | Egrandis_v1_0.052208m | Eucgr.E01565.1 | 18980927..18981637 | 5 |
| EgrERF-041 |  | Egrandis_v1_0.050019m | Eucgr.E01568.1 | 19043624..19044292 | 5 |
| EgrERF-042 |  | Egrandis_v1_0.050400m | Eucgr.E01569.1 | 19077327..19077995 | 5 |
| EgrERF-043 |  | Egrandis_v1_0.047527m | Eucgr.E01570.1 | 19100408..19101088 | 5 |
| EgrERF-044 |  | Egrandis_v1_0.044332m | Eucgr.E01571.1 | 19114440..19115036 | 5 |
| EgrERF-045 |  | Egrandis_v1_0.028443m | Eucgr.E02651.1 | 42424937..42425482 | 5 |
| EgrERF-046[P] |  | nd | nd | 42014641..42015179 | 5 |
| EgrERF-047 |  | Egrandis_v1_0.050841m | Eucgr.E02652.1 | 42439300..42440094 | 5 |
| EgrERF-048 |  | Egrandis_v1_0.043570m | Eucgr.E03167.1 | 53825206..53826000 | 5 |
| EgrERF-049 |  | Egrandis_v1_0.028452m | Eucgr.E03168.1 | 53840107..53840652 | 5 |
| EgrERF-050 |  | Egrandis_v1_0.024939m | Eucgr.F00659.1 | 8681782..8682545 | 6 |
| EgrERF-051 |  | Egrandis_v1_0.044165m | Eucgr.F01164.1 | 14952248..14952943 | 6 |
| EgrERF-052 |  | Egrandis_v1_0.014504m | Eucgr.F02691.1 | 36524215..36526180 | 6 |
| EgrERF-053 |  | nd | nd | 42067963..42068631 | 6 |
| EgrERF-054 |  | Egrandis_v1_0.025724m | Eucgr.F03499.1 | 43731346..43732023 | 6 |
| EgrERF-055 |  | Egrandis_v1_0.045622m | Eucgr.F03941.1 | 47764786..47765661 | 6 |
| EgrERF-056 |  | Egrandis_v1_0.027840m | Eucgr.F03947.1 | 47796310..47796984 | 6 |
| EgrERF-057 |  | Egrandis_v1_0.015515m | Eucgr.F04203.1 | 50260721..50264222 | 6 |
| EgrERF-058 |  | Egrandis_v1_0.046722m | Eucgr.G00002.1 | 55776..56549 | 7 |
| EgrERF-059[P] |  | Egrandis_v1_0.054506m | Eucgr.G00380.1 | 6852736..6853256 | 7 |
| EgrERF-060 |  | Egrandis_v1_0.043600m | Eucgr.G00382.1 | 6859315..6860169 | 7 |
| EgrERF-061 |  | Egrandis_v1_0.039539m | Eucgr.G00383.1 | 6863059..6863914 | 7 |
| EgrERF-062 |  | Egrandis_v1_0.052466m | Eucgr.G00384.1 | 6870328..6871182 | 7 |
| EgrERF-063 |  | Egrandis_v1_0.050355m | Eucgr.G00385.1 | 6877337..6878191 | 7 |
| EgrERF-064[P] |  | Egrandis_v1_0.045236m | Eucgr.G00386.1 | 6884603..6885588 | 7 |
| EgrERF-065 |  | Egrandis_v1_0.024653m | Eucgr.G00388.1 | 6897395..6898162 | 7 |
| EgrERF-066 |  | Egrandis_v1_0.042851m | Eucgr.G00390.1 | 6902873..6904145 | 7 |
| EgrERF-067 |  | Egrandis_v1_0.026787m | Eucgr.G00391.1 | 6925102..6925902 | 7 |
| EgrERF-068 |  | Egrandis_v1_0.051908m | Eucgr.G00392.1 | 6934254..6935084 | 7 |
| EgrERF-069 |  | Egrandis_v1_0.044682m | Eucgr.G00393.1 | 6947548..6948237 | 7 |
| EgrERF-070 |  | Egrandis_v1_0.019679m | Eucgr.G00394.1 | 6955739..6957134 | 7 |
| EgrERF-071 |  | Egrandis_v1_0.039297m | Eucgr.G00396.1 | 6983689..6984441 | 7 |
| EgrERF-072 |  | Egrandis_v1_0.027849m | Eucgr.G00397.1 | 6990344..6990968 | 7 |
| EgrERF-073 |  | Egrandis_v1_0.052498m | Eucgr.G00938.1 | 6947473..6948222 | 7 |
| EgrERF-074 |  | nd | nd | 16443662..16444139 | 7 |
| EgrERF-075 |  | Egrandis_v1_0.043191m | Eucgr.G00943.1 | 16485423..16485855 | 7 |
| EgrERF-076 |  | nd | nd | 24964732..24965580 | 7 |
| EgrERF-077 |  | Egrandis_v1_0.025764m | Eucgr.G01970.1 | 35737854..35741038 | 7 |
| EgrERF-078 |  | Egrandis_v1_0.019940m | Eucgr.G02326.1 | 41652646..41653599 | 7 |
| EgrERF-079 |  | Egrandis_v1_0.025138m | Eucgr.G02636.1 | 44680446..44681726 | 7 |
| EgrERF-080 |  | Egrandis_v1_0.010516m | Eucgr.G02793.1 | 46189726..46193180 | 7 |
| EgrERF-081 |  | Egrandis_v1_0.030841m | Eucgr.H00170.1 | 1713377..1713802 | 8 |
| EgrERF-082 |  | Egrandis_v1_0.054452m | Eucgr.H00172.1 | 1717208..1717675 | 8 |
| EgrERF-083 |  | Egrandis_v1_0.030854m | Eucgr.H00177.1 | 1763862..1764287 | 8 |
| EgrERF-084 |  | Egrandis_v1_0.020401m | Eucgr.H01085.1 | 13165596..13166615 | 8 |
| EgrERF-085 |  | Egrandis_v1_0.044642m | Eucgr.H01659.1 | 20291591..20292337 | 8 |
| EgrERF-086 |  | Egrandis_v1_0.043654m | Eucgr.H02495.1 | 34508368..34509387 | 8 |
| EgrERF-087 |  | Egrandis_v1_0.025437m | Eucgr.H03088.1 | 45359785..45360474 | 8 |
| EgrERF-088 |  | Egrandis_v1_0.050362m | Eucgr.H03090.1 | 45381214..45381651 | 8 |
| EgrERF-089 |  | Egrandis_v1_0.031018m | Eucgr.H03091.1 | 45424731..45425147 | 8 |
| EgrERF-090[P] |  | Egrandis_v1_0.053455m | Eucgr.H03365.1 | 49301537..49302027 | 8 |
| EgrERF-091 |  | Egrandis_v1_0.038205m | Eucgr.H03870.1 | 56480253..56481305 | 8 |
| EgrERF-092 |  | Egrandis_v1_0.024552m | Eucgr.H03965.1 | 57676445..57677578 | 8 |
| EgrERF-093 |  | Egrandis_v1_0.052040m | Eucgr.H04254.1 | 60672978..60673718 | 8 |
| EgrERF-094 |  | Egrandis_v1_0.022177m | Eucgr.H04892.1 | 69475161..69476641 | 8 |
| EgrERF-095 |  | Egrandis_v1_0.019103m | Eucgr.I00291.1 | 5685087..5686536 | 9 |
| EgrERF-096 |  | Egrandis_v1_0.040692m | Eucgr.I00292.1 | 5762446..5762970 | 9 |
| EgrERF-097 |  | Egrandis_v1_0.043578m | Eucgr.I01153.1 | 22624562..22625389 | 9 |
| EgrERF-098 |  | Egrandis_v1_0.040679m | Eucgr.I01576.1 | 25663687..25664445 | 9 |
| EgrERF-099 |  | Egrandis_v1_0.016576m | Eucgr.I02007.1 | 29741731..29742915 | 9 |
| EgrERF-100 |  | Egrandis_v1_0.053192m | Eucgr.I02155.1 | 31400032..31402809 | 9 |
| EgrERF-101 |  | Egrandis_v1_0.055033m | Eucgr.J01042.1 | 11393999..11395066 | 10 |
| EgrERF-102 |  | Egrandis_v1_0.022054m | Eucgr.K00126.1 | 1791834..1792688 | 11 |
| EgrERF-103 |  | Egrandis_v1_0.043025m | Eucgr.K00128.1 | 1796757..1797852 | 11 |
| EgrERF-104 |  | Egrandis_v1_0.019403m | Eucgr.K00321.1 | 3812531..3813610 | 11 |
| EgrERF-105 |  | Egrandis_v1_0.026173m | Eucgr.K00734.1 | 8491399..8492055 | 11 |
| EgrERF-106 |  | Egrandis_v1_0.023124m | Eucgr.K02193.1 | 29190709..29191723 | 11 |
| EgrERF-107 |  | Egrandis_v1_0.023161m | Eucgr.K02780.1 | 35879597..35880611 | 11 |
| EgrERF-108 |  | Egrandis_v1_0.024789m | Eucgr.K03266.1 | 41444331..41445053 | 11 |
|  |  |  |  |  |  |
| EgrRAV-01 |  | Egrandis_v1_0.016983m | Eucgr.B03048.1 | 55414093..55415184 | 2 |
| EgrRAV-02 |  | Egrandis_v1_0.054321m | Eucgr.B03049.1 | 55438194..55439177 | 2 |
| EgrRAV-03[P] |  | nd | nd | 55458507..55465107 | 2 |
| EgrRAV-04 |  | Egrandis_v1_0.050836m | Eucgr.B03644.1 | 60884976..60886352 | 2 |
| EgrRAV-05[P] |  | nd | nd | 60898756..60899514 | 2 |
| EgrRAV-06 |  | Egrandis_v1_0.018363m | Eucgr.C00358.1 | 6867710..6868735 | 3 |
| EgrRAV-07 |  | Egrandis_v1_0.054327m | Eucgr.D00053.1 | 754390..755400 | 4 |
| EgrRAV-08 |  | Egrandis_v1_0.042216m | Eucgr.G02345 | 41801648..41800590 | 7 |
|  |  |  |  |  |  |
| Soloist |  | Egrandis_v1_0.045497m | Eucgr.K02136.1 | 28504666..28509443 | 11 |

“Eucagen name” associates the subfamily name with the relative position on the scaffolds (1 to 11). “Generic name” given to EgrCBF(1-17) and EgrDREB2-(1-6) from literature uses and genome location.
